# Supplementary material for: Altered microRNA Transcriptome in Cultured Human Liver Cells upon Infection with Ebola Virus
Source: Int J Mol Sci. 2021 Apr 6;22(7):3792. doi: 10.3390/ijms22073792 (PMC8038836; doi:10.3390/ijms22073792)
Supplement: Supplementary file 1 [file ijms-22-03792-s001.zip › Supplementary_File/C_ GO_Analysis_Results/16-30nt_go_Reston-24h-Huh7_vs_Control-24h-Huh7_up.mature_mirna_targets/CC_result(Human).html]

| GO.ID | Term | Ontology | Count | Pop.Hits | List.Total | Pop.Total | Fold.Enrichment | Pvalue | FDR | Enrichment.Score | Gene.Ratio | GENES |
| --- | --- | --- | --- | --- | --- | --- | --- | --- | --- | --- | --- | --- |
| GO:0005622 | intracellular | Cellular component | 518 | 14673 | 596 | 18698 | 1.107540810245 | 5.22295386681923e-08 | 4.48651737159772e-05 | 7.28208381017188 | 0.869127516778524 | TRERF1//ZNF217//TBL1XR1//STX6//RTN3//FUT9//SERINC3//TMED10//CLCN5//RAB12//HS3ST5//STX12//HEPACAM2//GJA1//ARFIP1//RAB30//ST8SIA5//MAN1A1//ST8SIA3//RAB6B//RNF125//SYBU//CHST7//PMEPA1//ACBD3//GNPTG//CHST1//BLZF1//TNKS//DSEL//MMGT1//NDST3//UBE4B//DCUN1D4//NCK1//PPP1R15B//ZBTB18//SPO11//CDK6//CCNY//PLEKHF2//ULK2//CALCOCO2//ATG16L1//PIGP//PIGA//FMR1//SYCP3//SYCP1//DYNC1LI2//CLASP2//FBXO28//MTBP//CLIP1//CFDP1//DCTN6//CENPO//CEBPB//PTGES3//ATR//WRNIP1//NABP1//ESCO2//BAHD1//MAF//H3F3C//ESR1//IRF1//AR//POU4F1//RFX3//SMARCD2//RUNX3//DDX6//TEX11//SYCE1//PCGF5//VPS37A//CHMP3//CETN1//FAM110C//CALM2//CDC14A//MZT1//DCP2//PAN3//CNOT7//YTHDF2//CPEB1//RAI14//PAK6//DYNLL2//KRT27//SNRPN//SLC17A5//LDLR//NEU1//DRAM1//ARRDC3//RRAGD//SNX2//SOD1//CMC4//MIEF2//CCDC58//PRSS35//IMMP1L//ELK3//AK4//ACSL1//SLC44A1//MSRB3//GLRX//GLUD2//GPD1//COX8C//MACC1//JARID2//BOLA3//MDH1//MGST1//NOS1//DCAF8//MRPL35//XAF1//RDH14//METTL17//BNIP3//SOX4//UNG//DCTPP1//ABHD11//TACC2//IQGAP2//ZNF268//RAPGEF4//DNAJB4//STRAP//AKAP11//EGLN3//KLF12//ARAP2//ADH4//MTPN//UBXN2B//CRKL//SYT6//SH3D19//SPRED1//DENND1B//DCX//DLG2//DOK1//DPYSL2//PRSS55//EPS15//RASGEF1A//ZNRF2//RAB3GAP1//WDFY3//CDK19//EFR3A//OTUD3//DDHD2//DNAJC16//CNOT6L//LCLAT1//APPL1//TES//STEAP2//PTPN22//KLHL3//HSPB8//GJB2//SNX5//FAM126B//KIF4B//NOB1//GSTA2//GSTM3//HPRT1//AGFG1//HSBP1//IMPDH1//IRF2//KLC1//TNFAIP8L3//SULT6B1//ARHGAP1//MBNL1//MAP3K1//NR3C2//MTM1//MTRR//MYD88//ARHGEF4//MEMO1//RAB9B//PEX5L//CMPK1//PIK3CB//BRWD1//POLR2K//EGLN1//PPARG//MTMR10//PXK//CPPED1//PPP3CB//PRKAA1//ENAH//PRKAR2B//SPHK2//KCMF1//MAP3K7CL//DUSP22//CNOT6//ZBTB4//RAP2C//MID1IP1//MPP4//RRM2//ATXN1//SAMSN1//SRSF2//SH3BGR//SKP1//KRTAP4-11//MTMR9//SNAP25//SOS2//BTF3//TGFBR2//THOP1//TMOD1//TSC1//UBE2G1//VBP1//YES1//CA1//EVI5//SNIP1//UBA5//FOSL1//TRIM8//SBF2//CASP3//KBTBD8//C1ORF198//DGKE//EIF3B//CBL//LMLN//NAT1//CCT6A//NMI//LONRF1//AIMP1//DTD1//ARHGAP12//EI24//SOCS5//RAPGEF2//USP6NL//G3BP2//CDC27//ACBD5//MED6//TSHZ1//ANP32B//ZMYND11//TCERG1//KLF17//CPEB2//NCOA7//RNF38//ZNF782//GADD45A//CRISP1//ZNF800//ARX//CC2D1B//EMX2//EN1//TFEC//ZFP30//CPEB3//FOXJ3//FOXF2//PDZD2//MYT1L//RCOR1//ZFPM2//PTF1A//NUTM1//INTS7//GATA2//ATAD2//PURG//MDFIC//HIVEP2//HOXB6//SPOPL//ZNF680//ZNF568//MDM4//MEOX2//MLLT6//MYB//MYBL1//MYOG//NAP1L3//NEUROD1//NEUROG1//NFIA//NFE2//NHLH2//NPAT//NR4A2//OTX2//VGLL1//ARMCX3//RASD1//ARID4B//POU3F2//TET2//SMU1//UBE2W//STRBP//BTBD7//SERTAD4//SLC2A4RG//ZNF630//ZBTB26//MIER1//RBBP8//RFX4//DMRTA1//SH3BGRL//GMNC//RFX7//SLC30A5//BTG1//RHOXF2B//ZNF3//ZNF131//ZNF134//ZNF208//ZNF227//PTP4A1//ZNF655//BHLHE41//NPEPL1//CARF//FAM192A//TSC22D4//ARID5B//RHOXF2//NFATC2IP//ZIC5//BHLHE40//KLF7//RTCA//AKAP4//SNURF//RPS6KA5//PLAA//QKI//PHF14//ST18//RNF180//AKAP6//GPHN//IFI44//SIVA1//STMN2//SLAIN1//FRMD6//REG3G//SLC30A7//DGKH//EDN1//WDR47//CD2AP//FLRT1//GLRB//NRBF2//IGF1//RBM44//MET//DESI2//COMMD2//LGSN//ZFAND6//RALGPS2//PTGFR//HECW2//RBM25//RP2//BMPR2//SPOCK1//TBCA//FZD3//UBE3B//C5ORF30//DLG5//SLIT2//RAB28//RAD9B//PDIK1L//SCAF8//KDM2A//HBP1//HOXA3//HOXC8//HOXD1//KLF3//USP28//ZMAT3//LDLRAD4//MED28//ADAM12//LCOR//CBFB//BTAF1//WDR20//RBM41//NRXN1//MTX3//BCL2A1//GPD2//MICU3//SLC25A53//COX7A2//NLN//FASTK//PABPC5//CLCN6//LAMTOR3//CNTNAP2//LRP6//CDS1//VKORC1L1//SMIM14//SULF1//FIBIN//SHISA2//FAM19A1//IER3IP1//PLP2//STIM2//RTN1//MSMO1//ELOVL7//PLA2G12A//CAMLG//ARL6IP1//ERP27//ARSK//WNT7A//SLC35D1//HSD11B1//PIGS//FUT2//HEATR5A//GLT6D1//CCDC126//ITM2A//OGN//C16ORF70//C5AR1//PROCR//KIF13A//FAM92B//LRP8//SV2C//NTF3//TMEM63B//NPNT//FZD6//CHRM2//DBNDD2//PHACTR2//MMRN1//NHLRC2//VEGFB//DSC2//DLL1//CHIC1//EPC2//GRIK2//TDRD5//OSTF1//SLC2A5//IDS//SESTD1//AGTR2//INHBB//KCNS2//STK33//TNF//PSD//ENPP4//ADCYAP1//CSF2RA//EREG//NYAP1//ARL5A//IFNG//IL2//IL5//HPCAL4//TREM1//ARL15//CCL2//BTC//LONRF3//DIRAS3// |
| GO:0043226 | organelle | Cellular component | 482 | 13597 | 596 | 18698 | 1.11212303543073 | 1.67496652617938e-06 | 0.000550008672724882 | 5.7759938678157 | 0.808724832214765 | DYNLL2//RP2//GPHN//AKAP4//CALCOCO2//ARAP2//VPS37A//DENND1B//CC2D1B//EPS15//APPL1//GJA1//RAB30//AGFG1//NEU1//PPARG//RNF125//SYBU//SPHK2//PMEPA1//RBBP8//VBP1//LDLRAD4//EVI5//UBA5//RUNX3//BTAF1//SLC6A14//SLC30A7//STX12//GLT6D1//SNAP25//CALM2//NRXN1//OGN//DLG2//CPEB3//FMR1//NPTN//GRIK2//NOS1//PSD//CPEB1//BMPR2//BNIP3//SPOCK1//LRP8//DLG5//CMC4//MIEF2//CCDC58//DDX6//PRSS35//IMMP1L//ELK3//AK4//ACSL1//SLC44A1//MSRB3//RAI14//GLRX//GLUD2//GPD1//COX8C//MACC1//JARID2//BOLA3//MDH1//MGST1//DCAF8//MRPL35//XAF1//RDH14//METTL17//ACBD3//SOD1//SOX4//UNG//ZNF217//DCTPP1//ABHD11//AKAP11//ACBD5//MED6//TSHZ1//CDK6//UBE4B//ZBTB18//CEBPB//ANP32B//PTGES3//ZMYND11//ZNF268//TCERG1//EGLN3//ADH4//KLF17//CPEB2//NCOA7//MTPN//UBXN2B//RNF38//ESCO2//ZNF782//SPRED1//GADD45A//CRISP1//ZNF800//ARX//DOK1//EMX2//EN1//ESR1//CCNY//TFEC//ZFP30//FOXJ3//FOXF2//WDFY3//PDZD2//MYT1L//CDK19//DCUN1D4//RCOR1//ZFPM2//CNOT6L//PTF1A//NUTM1//INTS7//TES//PTPN22//GATA2//HSPB8//ATAD2//GSTM3//CNOT7//PURG//MDFIC//HIVEP2//HOXB6//HSBP1//SPOPL//ZNF680//IMPDH1//IRF1//AR//ZNF568//MAF//MBNL1//MDM4//MEOX2//MLLT6//H3F3C//MYB//MYBL1//MYD88//MYOG//NAP1L3//NCK1//NEUROD1//NEUROG1//NFIA//NFE2//NHLH2//NPAT//NR4A2//OTX2//SYCP3//MEMO1//YTHDF2//VGLL1//ARMCX3//RASD1//CMPK1//ARID4B//PIK3CB//BRWD1//POLR2K//ATR//POU3F2//POU4F1//EGLN1//TET2//SMU1//UBE2W//STRBP//PRKAA1//BTBD7//TRERF1//SERTAD4//SLC2A4RG//WRNIP1//MAP3K7CL//PAK6//DUSP22//ZNF630//CNOT6//ZBTB4//ZBTB26//MIER1//MID1IP1//RRAGD//RFX3//RFX4//ATXN1//DMRTA1//SAMSN1//FAM110C//SRSF2//SH3BGRL//GMNC//NABP1//RFX7//SLC30A5//SKP1//BTF3//BTG1//TSC1//RHOXF2B//ZNF3//ZNF131//ZNF134//ZNF208//ZNF227//PTP4A1//ZNF655//BHLHE41//NPEPL1//TBL1XR1//SNIP1//CARF//FAM192A//FOSL1//TSC22D4//CASP3//ARID5B//PCGF5//RHOXF2//NFATC2IP//DGKE//ZIC5//BLZF1//BHLHE40//CDC14A//KLF7//RTCA//TNKS//CBL//SNURF//RPS6KA5//AIMP1//PLAA//QKI//PHF14//ST18//CDC27//RAB12//NRBF2//ATG16L1//PLEKHF2//RTN3//CDS1//TMED10//VKORC1L1//SMIM14//SULF1//LCLAT1//FIBIN//SHISA2//LRP6//FAM19A1//MAN1A1//IER3IP1//PLP2//STIM2//RTN1//MSMO1//ELOVL7//PLA2G12A//CAMLG//PPP1R15B//EI24//DDHD2//GJB2//RAB6B//STX6//FUT9//STMN2//CLCN5//RAB3GAP1//CLASP2//FUT2//HEPACAM2//HEATR5A//CNTNAP2//LDLR//YES1//KBTBD8//GNPTG//CCDC126//MMGT1//ITM2A//AKAP6//DSC2//FLRT1//DLL1//KLC1//NTF3//CHMP3//CHIC1//USP6NL//PROCR//IQGAP2//DNAJB4//VSIG4//GGT6//COX7A2//CRKL//A2ML1//DPYSL2//COBLL1//ENPP4//EFR3A//CD2AP//NPNT//OSTF1//IAH1//GSTA2//HPRT1//KRT27//ARHGAP1//MEP1A//MTM1//PGC//ARL15//CCDC25//CPPED1//PRKAR2B//CCL28//RAP2C//PTPRG//SLC1A1//BMP3//SLC2A5//SNX2//TBCA//UBE2G1//WNT7A//CA1//MMRN2//LAMTOR3//EIF3B//SLC4A4//CD84//CCT6A//SLIT2//DYNC1LI2//FBXO28//MTBP//CLIP1//ZMAT3//DTD1//LMLN//CETN1//FAM92B//MZT1//SLAIN1//FRMD6//DCX//KLHL3//ENAH//TMOD1//SPO11//CFDP1//DCTN6//CENPO//TMEM63B//MPP4//TACC2//HOXC8//DCP2//SESTD1//HECW2//DGKH//STEAP2//SNX5//ARRDC3//EDN1//RAB9B//RAPGEF2//SLC17A5//DRAM1//SYCP1//TNF//PEX5L//SV2C//IGF1//MED28//PAN3//TDRD5//RAD9B//CLCN6//ZNRF2//IDS//C5AR1//SYT6//DBNDD2//C16ORF70//PXK//WDR47//KIF4B//KIF13A//CHRM2//PHACTR2//MMRN1//NHLRC2//VEGFB//KCMF1//SERINC3//HS3ST5//ARFIP1//ST8SIA5//ST8SIA3//CHST7//CHST1//DSEL//NDST3//PIGP//PIGA//BAHD1//SMARCD2//TEX11//SYCE1//SNRPN//RNF180//SIVA1//STRAP//KLF12//PDIK1L//SH3D19//SCAF8//KDM2A//HBP1//NOB1//HOXA3//HOXD1//IRF2//TNFAIP8L3//NR3C2//KLF3//PPP3CB//USP28//ADAM12//LCOR//CBFB//NMI//WDR20//RBM25//RBM41//MTX3//BCL2A1//GPD2//MICU3//SLC25A53//NLN//THOP1//FASTK//PABPC5//SBF2//ARL6IP1//ERP27//ARSK//SLC35D1//HSD11B1//PIGS//TRIM8//FZD6//ULK2//EPC2//RAB28//C5ORF30//KRTAP4-11// |
| GO:0043229 | intracellular organelle | Cellular component | 453 | 12604 | 596 | 18698 | 1.12755751343453 | 1.92086847284592e-06 | 0.000550008672724882 | 5.7165023714594 | 0.76006711409396 | CALCOCO2//ARAP2//VPS37A//DENND1B//CC2D1B//EPS15//APPL1//GJA1//RAB30//AGFG1//NEU1//PPARG//RNF125//SYBU//SPHK2//PMEPA1//RBBP8//VBP1//LDLRAD4//EVI5//UBA5//RUNX3//BTAF1//CMC4//MIEF2//CCDC58//DYNLL2//DDX6//PRSS35//IMMP1L//ELK3//AK4//ACSL1//SLC44A1//MSRB3//RAI14//GLRX//GLUD2//GPD1//COX8C//MACC1//JARID2//BOLA3//MDH1//MGST1//NOS1//DCAF8//MRPL35//XAF1//RDH14//METTL17//ACBD3//BNIP3//SOD1//SOX4//UNG//ZNF217//DCTPP1//ABHD11//MED6//TSHZ1//CDK6//UBE4B//ZBTB18//CEBPB//ANP32B//PTGES3//ZMYND11//ZNF268//TCERG1//EGLN3//ADH4//KLF17//CPEB2//NCOA7//MTPN//UBXN2B//RNF38//ESCO2//ZNF782//SPRED1//GADD45A//CRISP1//ZNF800//ARX//DOK1//EMX2//EN1//ESR1//CCNY//TFEC//ZFP30//CPEB3//FOXJ3//FOXF2//WDFY3//PDZD2//MYT1L//CDK19//DCUN1D4//RCOR1//FMR1//ZFPM2//CNOT6L//PTF1A//NUTM1//INTS7//TES//PTPN22//GATA2//HSPB8//ATAD2//GSTM3//CNOT7//PURG//MDFIC//HIVEP2//HOXB6//HSBP1//SPOPL//ZNF680//IMPDH1//IRF1//AR//ZNF568//MAF//MBNL1//MDM4//MEOX2//MLLT6//H3F3C//MYB//MYBL1//MYD88//MYOG//NAP1L3//NCK1//NEUROD1//NEUROG1//NFIA//NFE2//NHLH2//NPAT//NR4A2//OTX2//SYCP3//MEMO1//YTHDF2//VGLL1//ARMCX3//RASD1//CMPK1//ARID4B//PIK3CB//BRWD1//POLR2K//ATR//POU3F2//POU4F1//EGLN1//TET2//SMU1//UBE2W//STRBP//PRKAA1//BTBD7//TRERF1//SERTAD4//SLC2A4RG//WRNIP1//MAP3K7CL//PAK6//DUSP22//ZNF630//CNOT6//ZBTB4//ZBTB26//MIER1//MID1IP1//RRAGD//RFX3//RFX4//ATXN1//DMRTA1//SAMSN1//FAM110C//SRSF2//CPEB1//SH3BGRL//GMNC//NABP1//RFX7//SLC30A5//SKP1//BTF3//BTG1//TSC1//RHOXF2B//ZNF3//ZNF131//ZNF134//ZNF208//ZNF227//PTP4A1//ZNF655//BHLHE41//NPEPL1//TBL1XR1//SNIP1//CARF//FAM192A//CALM2//FOSL1//TSC22D4//CASP3//ARID5B//PCGF5//RHOXF2//NFATC2IP//DGKE//ZIC5//BLZF1//BHLHE40//CDC14A//KLF7//RTCA//TNKS//CBL//AKAP4//SNURF//ACBD5//RPS6KA5//AIMP1//PLAA//QKI//PHF14//ST18//CDC27//RTN3//CDS1//TMED10//VKORC1L1//SMIM14//SULF1//LCLAT1//FIBIN//SHISA2//LRP6//FAM19A1//MAN1A1//IER3IP1//PLP2//STIM2//RTN1//MSMO1//PLEKHF2//ELOVL7//PLA2G12A//CAMLG//PPP1R15B//NRXN1//EI24//DDHD2//GJB2//RAB6B//STX6//FUT9//STMN2//CLCN5//SLC30A7//RAB12//RAB3GAP1//CLASP2//FUT2//HEPACAM2//HEATR5A//CNTNAP2//GLT6D1//LDLR//RP2//YES1//KBTBD8//GNPTG//CCDC126//MMGT1//ITM2A//DYNC1LI2//FBXO28//MTBP//CLIP1//AKAP11//ZMAT3//TBCA//DTD1//LMLN//CETN1//FAM92B//MZT1//GPHN//SLAIN1//FRMD6//DCX//KLHL3//ENAH//SNAP25//TMOD1//DSC2//FLRT1//DLL1//NRBF2//KLC1//NTF3//CHMP3//CHIC1//USP6NL//CFDP1//DCTN6//CENPO//NPNT//SLC17A5//DRAM1//ARRDC3//SNX2//ATG16L1//SYCP1//SPO11//AKAP6//HECW2//IQGAP2//CD2AP//TMEM63B//MPP4//TACC2//HOXC8//MED28//SESTD1//CRKL//DGKH//STEAP2//SNX5//MTM1//RAPGEF2//EDN1//RAB9B//STX12//TNF//IGF1//ARHGAP1//DCP2//PAN3//TDRD5//PEX5L//SV2C//RAD9B//CLCN6//ZNRF2//LAMTOR3//IDS//OGN//C5AR1//OSTF1//WNT7A//SYT6//DBNDD2//C16ORF70//PXK//DPYSL2//WDR47//KIF4B//KIF13A//CCT6A//LRP8//CHRM2//PHACTR2//MMRN1//NHLRC2//VEGFB//SLC2A5//CPPED1//KRT27//RAP2C//ENPP4//KCMF1//SERINC3//HS3ST5//ARFIP1//ST8SIA5//ST8SIA3//CHST7//CHST1//DSEL//NDST3//PIGP//PIGA//BAHD1//SMARCD2//TEX11//SYCE1//SNRPN//RNF180//SIVA1//DNAJB4//STRAP//KLF12//PDIK1L//SH3D19//SCAF8//KDM2A//HBP1//NOB1//HOXA3//HOXD1//IRF2//TNFAIP8L3//NR3C2//KLF3//PPP3CB//USP28//BMPR2//ADAM12//LCOR//CBFB//NMI//WDR20//RBM25//RBM41//MTX3//BCL2A1//GPD2//MICU3//SLC25A53//PRKAR2B//COX7A2//NLN//THOP1//FASTK//PABPC5//SBF2//ARL6IP1//ERP27//ARSK//SLC35D1//HSD11B1//PIGS//PROCR//TRIM8//FZD6//ULK2//EPC2//RAB28//DLG5//KRTAP4-11//PSD// |
| GO:0044424 | intracellular part | Cellular component | 503 | 14395 | 596 | 18698 | 1.09623890659275 | 3.48787722740256e-06 | 0.0007490216345847 | 5.4574388105514 | 0.843959731543624 | UBE4B//DCUN1D4//CDK6//CALCOCO2//GPHN//CEBPB//ANP32B//IFI44//SIVA1//TACC2//PTGES3//IQGAP2//ZNF268//STMN2//STRAP//AKAP11//EGLN3//ARAP2//SLAIN1//FRMD6//REG3G//CPEB2//SLC30A7//DGKH//GADD45A//DDX6//DCP2//EDN1//ESR1//CCNY//ZNRF2//TFEC//CPEB3//WDR47//WDFY3//PDZD2//CLASP2//OTUD3//DDHD2//FMR1//ZFPM2//CD2AP//FLRT1//CNOT6L//PTF1A//NUTM1//INTS7//APPL1//PTPN22//HSPB8//ARFIP1//GLRB//GSTM3//CNOT7//MDFIC//NRBF2//HPRT1//AGFG1//HSBP1//SPOPL//MACC1//IGF1//IMPDH1//IRF1//AR//RBM44//TNFAIP8L3//ARHGAP1//MAF//MBNL1//MAP3K1//MEOX2//MET//MTM1//MYD88//NCK1//NEU1//NEUROD1//NFE2//NOS1//NPAT//NR4A2//DCAF8//DESI2//MEMO1//COMMD2//LGSN//CMPK1//ARID4B//PIK3CB//ZFAND6//POU4F1//EGLN1//MTMR10//PXK//RALGPS2//SMU1//UBE2W//PPP3CB//DRAM1//STRBP//PRKAA1//PRKAR2B//TEX11//SLC2A4RG//PAK6//DUSP22//PTGFR//HECW2//MIER1//RAP2C//RBM25//RRAGD//RP2//ATXN1//SAMSN1//SH3BGR//CPEB1//SH3BGRL//SKP1//BMPR2//MTMR9//SNAP25//SNRPN//BNIP3//SNX2//SOD1//SOX4//SPOCK1//TBCA//BTG1//TSC1//UBE2G1//VBP1//PTP4A1//ZNF655//NPEPL1//FZD3//UBA5//CALM2//TSC22D4//CASP3//NFATC2IP//PPP1R15B//DGKE//BLZF1//BHLHE40//CDC14A//RUNX3//TNKS//AKAP4//UBE3B//C5ORF30//CCT6A//NMI//DLG5//RPS6KA5//DTD1//SLIT2//RAB28//PLAA//QKI//AKAP6//SOCS5//RAPGEF2//G3BP2//CDC27//FOXF2//RCOR1//GATA2//MYOG//NHLH2//POU3F2//TRERF1//RFX3//AIMP1//CNOT6//SNX5//YES1//EPS15//GPD2//MICU3//SLC25A53//MRPL35//MIEF2//ACSL1//SLC44A1//GJA1//MTX3//MGST1//BCL2A1//JARID2//VPS37A//DENND1B//CC2D1B//RAB30//PPARG//RNF125//SYBU//SPHK2//PMEPA1//RBBP8//LDLRAD4//EVI5//BTAF1//STX12//PLEKHF2//ULK2//ATG16L1//CMC4//CCDC58//DYNLL2//PRSS35//IMMP1L//ELK3//AK4//MSRB3//RAI14//GLRX//GLUD2//GPD1//COX8C//BOLA3//MDH1//XAF1//RDH14//METTL17//ACBD3//UNG//ZNF217//DCTPP1//ABHD11//STX6//DCTN6//RAPGEF4//DNAJB4//KLF12//CLCN5//ADH4//MTPN//UBXN2B//CRKL//SYT6//SH3D19//SPRED1//DCX//DLG2//DYNC1LI2//DOK1//DPYSL2//RAB12//PRSS55//RASGEF1A//RAB3GAP1//CDK19//EFR3A//DNAJC16//LCLAT1//PAN3//TES//STEAP2//KLHL3//SLC17A5//GJB2//FAM126B//KIF4B//NOB1//GSTA2//KRT27//IRF2//KLC1//SULT6B1//MAN1A1//NR3C2//MZT1//MTRR//ARHGEF4//RAB9B//YTHDF2//PEX5L//RAB6B//CHMP3//BRWD1//POLR2K//CPPED1//ENAH//KCMF1//MAP3K7CL//ZBTB4//MID1IP1//MPP4//RRM2//CLIP1//SRSF2//NABP1//KRTAP4-11//SOS2//BTF3//TGFBR2//THOP1//TMOD1//CA1//CENPO//SNIP1//FOSL1//TRIM8//SBF2//KBTBD8//C1ORF198//EIF3B//CBL//LMLN//NAT1//LONRF1//ARHGAP12//EI24//USP6NL//RTN3//CDS1//TMED10//VKORC1L1//SMIM14//SULF1//FIBIN//SHISA2//LRP6//FAM19A1//IER3IP1//PLP2//STIM2//RTN1//MSMO1//ELOVL7//PLA2G12A//CAMLG//NRXN1//FUT9//ESCO2//FUT2//HEPACAM2//HEATR5A//CNTNAP2//GLT6D1//LDLR//ATR//SLC30A5//GNPTG//CCDC126//MMGT1//ITM2A//DSC2//DLL1//NTF3//CHIC1//SERINC3//AGTR2//INHBB//KCNS2//RASD1//WRNIP1//STK33//FASTK//PABPC5//ERP27//ARSK//WNT7A//OGN//OSTF1//NPNT//ARRDC3//FAM110C//IDS//ACBD5//MED6//TSHZ1//ZBTB18//ZMYND11//TCERG1//KLF17//NCOA7//RNF38//ZNF782//CRISP1//ZNF800//ARX//EMX2//EN1//ZFP30//FOXJ3//MYT1L//ATAD2//PURG//HIVEP2//HOXB6//ZNF680//ZNF568//MDM4//MLLT6//H3F3C//MYB//MYBL1//NAP1L3//NEUROG1//NFIA//OTX2//SYCP3//VGLL1//ARMCX3//TET2//BTBD7//SERTAD4//ZNF630//ZBTB26//RFX4//DMRTA1//GMNC//RFX7//RHOXF2B//ZNF3//ZNF131//ZNF134//ZNF208//ZNF227//BHLHE41//TBL1XR1//CARF//FAM192A//ARID5B//PCGF5//RHOXF2//ZIC5//KLF7//RTCA//SNURF//PHF14//ST18//PSD//TNF//RNF180//NLN//C16ORF70//TDRD5//FBXO28//MTBP//ZMAT3//CETN1//FAM92B//SPO11//CFDP1//TMEM63B//HOXC8//SESTD1//SYCP1//BAHD1//SMARCD2//SYCE1//RAD9B//PDIK1L//SCAF8//KDM2A//HBP1//HOXA3//HOXD1//KLF3//USP28//MED28//ADAM12//LCOR//CBFB//WDR20//RBM41//COX7A2//PROCR//KIF13A//LRP8//HS3ST5//ST8SIA5//ST8SIA3//CHST7//CHST1//DSEL//NDST3//PIGP//PIGA//ARL6IP1//SLC35D1//HSD11B1//PIGS//C5AR1//CLCN6//LAMTOR3//FZD6//MMRN1//NHLRC2//VEGFB//EPC2//GRIK2//SV2C//DBNDD2//PHACTR2//SLC2A5//ENPP4//CHRM2// |
| GO:0043227 | membrane-bounded organelle | Cellular component | 451 | 12616 | 596 | 18698 | 1.12151156728645 | 5.4352387783575e-06 | 0.000933774022121819 | 5.26478137193736 | 0.756711409395973 | CALCOCO2//ARAP2//VPS37A//DENND1B//CC2D1B//EPS15//APPL1//GJA1//RAB30//AGFG1//NEU1//PPARG//RNF125//SYBU//SPHK2//PMEPA1//RBBP8//VBP1//LDLRAD4//EVI5//UBA5//RUNX3//BTAF1//SLC6A14//SLC30A7//STX12//GLT6D1//SNAP25//CALM2//NRXN1//CMC4//MIEF2//CCDC58//DYNLL2//DDX6//PRSS35//IMMP1L//ELK3//AK4//ACSL1//SLC44A1//MSRB3//RAI14//GLRX//GLUD2//GPD1//COX8C//MACC1//JARID2//BOLA3//MDH1//MGST1//NOS1//DCAF8//MRPL35//XAF1//RDH14//METTL17//ACBD3//BNIP3//SOD1//SOX4//UNG//ZNF217//DCTPP1//ABHD11//MED6//TSHZ1//CDK6//UBE4B//ZBTB18//CEBPB//ANP32B//PTGES3//ZMYND11//ZNF268//TCERG1//EGLN3//ADH4//KLF17//CPEB2//NCOA7//MTPN//UBXN2B//RNF38//ESCO2//ZNF782//SPRED1//GADD45A//CRISP1//ZNF800//ARX//DOK1//EMX2//EN1//ESR1//CCNY//TFEC//ZFP30//CPEB3//FOXJ3//FOXF2//WDFY3//PDZD2//MYT1L//CDK19//DCUN1D4//RCOR1//FMR1//ZFPM2//CNOT6L//PTF1A//NUTM1//INTS7//TES//PTPN22//GATA2//HSPB8//ATAD2//GSTM3//CNOT7//PURG//MDFIC//HIVEP2//HOXB6//HSBP1//SPOPL//ZNF680//IMPDH1//IRF1//AR//ZNF568//MAF//MBNL1//MDM4//MEOX2//MLLT6//H3F3C//MYB//MYBL1//MYD88//MYOG//NAP1L3//NCK1//NEUROD1//NEUROG1//NFIA//NFE2//NHLH2//NPAT//NR4A2//OTX2//SYCP3//MEMO1//YTHDF2//VGLL1//ARMCX3//RASD1//CMPK1//ARID4B//PIK3CB//BRWD1//POLR2K//ATR//POU3F2//POU4F1//EGLN1//TET2//SMU1//UBE2W//STRBP//PRKAA1//BTBD7//TRERF1//SERTAD4//SLC2A4RG//WRNIP1//MAP3K7CL//PAK6//DUSP22//ZNF630//CNOT6//ZBTB4//ZBTB26//MIER1//MID1IP1//RRAGD//RFX3//RFX4//ATXN1//DMRTA1//SAMSN1//FAM110C//SRSF2//CPEB1//SH3BGRL//GMNC//NABP1//RFX7//SLC30A5//SKP1//BTF3//BTG1//TSC1//RHOXF2B//ZNF3//ZNF131//ZNF134//ZNF208//ZNF227//PTP4A1//ZNF655//BHLHE41//NPEPL1//TBL1XR1//SNIP1//CARF//FAM192A//FOSL1//TSC22D4//CASP3//ARID5B//PCGF5//RHOXF2//NFATC2IP//DGKE//ZIC5//BLZF1//BHLHE40//CDC14A//KLF7//RTCA//TNKS//CBL//AKAP4//SNURF//ACBD5//RPS6KA5//AIMP1//PLAA//QKI//PHF14//ST18//CDC27//RTN3//CDS1//TMED10//VKORC1L1//SMIM14//SULF1//LCLAT1//FIBIN//SHISA2//LRP6//FAM19A1//MAN1A1//IER3IP1//PLP2//STIM2//RTN1//MSMO1//PLEKHF2//ELOVL7//PLA2G12A//CAMLG//PPP1R15B//EI24//DDHD2//GJB2//RAB6B//STX6//FUT9//STMN2//CLCN5//RAB12//RAB3GAP1//CLASP2//FUT2//HEPACAM2//HEATR5A//CNTNAP2//LDLR//RP2//YES1//KBTBD8//GNPTG//CCDC126//MMGT1//ITM2A//OGN//DYNC1LI2//SLC17A5//DRAM1//ARRDC3//SNX2//NRBF2//ATG16L1//SYCP1//AKAP6//AKAP11//DSC2//FLRT1//DLL1//KLC1//NTF3//CHMP3//CHIC1//USP6NL//PROCR//IQGAP2//DNAJB4//VSIG4//GGT6//COX7A2//CRKL//A2ML1//DPYSL2//COBLL1//ENPP4//EFR3A//CD2AP//NPNT//OSTF1//IAH1//GSTA2//HPRT1//KRT27//ARHGAP1//MEP1A//MTM1//PGC//ARL15//CCDC25//CPPED1//PRKAR2B//CCL28//RAP2C//PTPRG//SLC1A1//BMP3//SLC2A5//TBCA//UBE2G1//WNT7A//CA1//MMRN2//LAMTOR3//EIF3B//SLC4A4//CD84//CCT6A//SLIT2//PEX5L//DGKH//STEAP2//SNX5//CLIP1//EDN1//RAB9B//RAPGEF2//TNF//SV2C//IGF1//CLCN6//ZNRF2//IDS//C5AR1//SYT6//DBNDD2//C16ORF70//CHRM2//PHACTR2//MMRN1//NHLRC2//VEGFB//KCMF1//SERINC3//HS3ST5//ARFIP1//ST8SIA5//ST8SIA3//CHST7//CHST1//DSEL//NDST3//MTX3//BCL2A1//GPD2//MICU3//SLC25A53//SBF2//KIF13A//FZD6//ULK2//RNF180//NLN//THOP1//FASTK//PABPC5//SPO11//SMARCD2//TEX11//SYCE1//SNRPN//SIVA1//TACC2//STRAP//KLF12//RAD9B//PDIK1L//SH3D19//DCP2//SCAF8//BAHD1//KDM2A//HBP1//KIF4B//NOB1//HOXA3//HOXC8//HOXD1//IRF2//TNFAIP8L3//NR3C2//KLF3//PPP3CB//USP28//ZMAT3//BMPR2//CENPO//MED28//ADAM12//LCOR//CBFB//NMI//WDR20//RBM25//RBM41//DTD1//TMOD1//TRIM8//EPC2//PSD//PIGP//PIGA//ARL6IP1//ERP27//ARSK//SLC35D1//HSD11B1//PIGS// |
| GO:0043231 | intracellular membrane-bounded organelle | Cellular component | 390 | 10864 | 596 | 18698 | 1.12622132330411 | 0.000117133822533053 | 0.0167696589259821 | 3.93131768376162 | 0.654362416107383 | CMC4//MIEF2//CCDC58//DYNLL2//DDX6//PRSS35//IMMP1L//ELK3//AK4//ACSL1//SLC44A1//MSRB3//RAI14//GJA1//GLRX//GLUD2//GPD1//COX8C//MACC1//JARID2//BOLA3//MDH1//MGST1//NOS1//DCAF8//MRPL35//XAF1//RDH14//METTL17//ACBD3//BNIP3//SOD1//SOX4//UNG//ZNF217//DCTPP1//ABHD11//MED6//TSHZ1//CDK6//CALCOCO2//UBE4B//ZBTB18//CEBPB//ANP32B//PTGES3//ZMYND11//ZNF268//TCERG1//EGLN3//ADH4//KLF17//CPEB2//NCOA7//MTPN//UBXN2B//RNF38//ESCO2//ZNF782//SPRED1//GADD45A//CRISP1//ZNF800//ARX//DOK1//CC2D1B//EMX2//EN1//ESR1//CCNY//TFEC//ZFP30//CPEB3//FOXJ3//FOXF2//WDFY3//PDZD2//MYT1L//CDK19//DCUN1D4//RCOR1//FMR1//ZFPM2//CNOT6L//PTF1A//NUTM1//INTS7//APPL1//TES//PTPN22//GATA2//HSPB8//ATAD2//GSTM3//CNOT7//PURG//MDFIC//HIVEP2//HOXB6//HSBP1//SPOPL//ZNF680//IMPDH1//IRF1//AR//ZNF568//MAF//MBNL1//MDM4//MEOX2//MLLT6//H3F3C//MYB//MYBL1//MYD88//MYOG//NAP1L3//NCK1//NEUROD1//NEUROG1//NFIA//NFE2//NHLH2//NPAT//NR4A2//OTX2//SYCP3//MEMO1//YTHDF2//VGLL1//ARMCX3//RASD1//CMPK1//ARID4B//PIK3CB//BRWD1//POLR2K//ATR//POU3F2//POU4F1//EGLN1//PPARG//TET2//SMU1//UBE2W//STRBP//PRKAA1//BTBD7//TRERF1//SERTAD4//SLC2A4RG//WRNIP1//MAP3K7CL//PAK6//DUSP22//ZNF630//CNOT6//ZBTB4//ZBTB26//MIER1//MID1IP1//RRAGD//RBBP8//RFX3//RFX4//ATXN1//DMRTA1//SAMSN1//FAM110C//SRSF2//CPEB1//SH3BGRL//GMNC//NABP1//RFX7//SLC30A5//SKP1//BTF3//BTG1//TSC1//RHOXF2B//VBP1//ZNF3//ZNF131//ZNF134//ZNF208//ZNF227//PTP4A1//EVI5//ZNF655//BHLHE41//NPEPL1//TBL1XR1//SNIP1//CARF//UBA5//FAM192A//CALM2//FOSL1//TSC22D4//CASP3//ARID5B//PCGF5//RHOXF2//NFATC2IP//DGKE//ZIC5//BLZF1//BHLHE40//CDC14A//KLF7//RTCA//RUNX3//TNKS//CBL//AKAP4//SNURF//ACBD5//RPS6KA5//AIMP1//PLAA//QKI//PHF14//ST18//CDC27//RTN3//CDS1//TMED10//VKORC1L1//SMIM14//SULF1//LCLAT1//FIBIN//SHISA2//LRP6//FAM19A1//MAN1A1//IER3IP1//PLP2//STIM2//RTN1//MSMO1//PLEKHF2//ELOVL7//PLA2G12A//CAMLG//PPP1R15B//NRXN1//EI24//DDHD2//GJB2//RAB6B//STX6//FUT9//STMN2//CLCN5//SLC30A7//RAB12//RAB3GAP1//CLASP2//FUT2//HEPACAM2//HEATR5A//CNTNAP2//GLT6D1//LDLR//RP2//YES1//KBTBD8//GNPTG//CCDC126//MMGT1//ITM2A//RAB30//NRBF2//ATG16L1//AKAP6//AKAP11//DYNC1LI2//SLC17A5//NEU1//DRAM1//ARRDC3//SNX2//SYCP1//PEX5L//CLCN6//ZNRF2//SPHK2//LAMTOR3//IDS//OGN//EDN1//CPPED1//MTX3//BCL2A1//GPD2//MICU3//SLC25A53//PRKAR2B//COX7A2//NLN//THOP1//FASTK//PABPC5//SBF2//SPO11//SMARCD2//TEX11//SYCE1//SNRPN//RNF180//CLIP1//AGFG1//SIVA1//TACC2//DNAJB4//STRAP//KLF12//VPS37A//CRKL//RAD9B//PDIK1L//SH3D19//DCP2//SCAF8//BAHD1//KDM2A//HBP1//KIF4B//NOB1//HOXA3//HOXC8//HOXD1//IRF2//TNFAIP8L3//NR3C2//KLF3//PPP3CB//USP28//ZMAT3//BMPR2//LDLRAD4//CENPO//MED28//ADAM12//LCOR//CBFB//BTAF1//NMI//WDR20//RBM25//RBM41//TBCA//DTD1//TMOD1//TRIM8//DENND1B//EPC2//PIGP//PIGA//ARL6IP1//ERP27//ARSK//WNT7A//SLC35D1//FLRT1//HSD11B1//PIGS//RNF125//SYT6//SERINC3//HS3ST5//STX12//ARFIP1//ST8SIA5//ST8SIA3//SYBU//CHST7//PMEPA1//CHST1//DSEL//NDST3//SNAP25//C16ORF70//STEAP2//KIF13A//USP6NL//ARAP2//EPS15// |
| GO:0098805 | whole membrane | Cellular component | 76 | 1630 | 596 | 18698 | 1.46276608885412 | 0.0004857382308487 | 0.059607020042719 | 3.31359771330026 | 0.12751677852349 | PLEKHF2//SBF2//IQGAP2//TMED10//RAB9B//SLC30A5//C5AR1//CLCN5//CLCN6//VPS37A//ZNRF2//APPL1//STEAP2//ARHGAP1//LDLR//MYD88//PMEPA1//KIF13A//SNX2//LAMTOR3//GJA1//WNT7A//ATG16L1//ULK2//CALCOCO2//PHACTR2//MGST1//SLC2A5//SNAP25//RAP2C//ENPP4//EPS15//STX12//SNX5//LRP6//LDLRAD4//MMGT1//CHMP3//RAB12//CHRM2//ACSL1//PEX5L//ACBD5//MIEF2//SLC44A1//MTX3//BCL2A1//BNIP3//SLC17A5//NEU1//DRAM1//SPHK2//RDH14//SYT6//SV2C//DBNDD2//C16ORF70//WDFY3//RRAGD//PSD//SPRED1//EFNA5//NOS1//BMPR2//TGFBR2//LRP8//AKAP6//CD8A//SULF1//DLL1//PRKAR2B//TNF//CASP3//CBL//ARMCX3//STX6// |
| GO:0005634 | nucleus | Cellular component | 270 | 7267 | 596 | 18698 | 1.16562136642337 | 0.000663147366281166 | 0.0712054484544402 | 3.17838995085459 | 0.453020134228188 | SYCP1//TRERF1//ZNF217//TBL1XR1//ZBTB18//SPO11//ATR//WRNIP1//NABP1//TNKS//CEBPB//ESR1//IRF1//AR//POU4F1//RFX3//SMARCD2//RUNX3//SYCP3//TEX11//SYCE1//RAI14//PAK6//SNRPN//WDFY3//RNF180//CLIP1//BNIP3//AKAP6//AGFG1//MED6//CDK6//STX6//SIVA1//TACC2//PTGES3//ZMYND11//TCERG1//DNAJB4//STRAP//EGLN3//KLF12//VPS37A//CRKL//RAD9B//PDIK1L//RNF38//SH3D19//ESCO2//GADD45A//DCP2//CC2D1B//ELK3//TFEC//SCAF8//BAHD1//KDM2A//RCOR1//FMR1//ZFPM2//SLC44A1//INTS7//GATA2//HSPB8//HBP1//GJA1//KIF4B//NOB1//ATAD2//NRBF2//HIVEP2//HOXA3//HOXC8//HOXD1//HSBP1//IRF2//JARID2//TNFAIP8L3//MBNL1//MDM4//NR3C2//MYB//MYOG//NEUROD1//NFIA//NFE2//NOS1//NPAT//NR4A2//DCAF8//KLF3//ARID4B//BRWD1//POLR2K//PPARG//UBE2W//PPP3CB//PRKAA1//USP28//ZBTB4//MIER1//RRAGD//RBBP8//RP2//ATXN1//SRSF2//ZMAT3//CPEB1//METTL17//SKP1//BMPR2//SOD1//SOX4//UNG//LDLRAD4//ZNF131//CENPO//SNIP1//CARF//MED28//ADAM12//FOSL1//CASP3//ARID5B//PCGF5//LCOR//CDC14A//RTCA//CBFB//BTAF1//NMI//ACBD5//WDR20//RPS6KA5//CDC27//RBM25//RBM41//H3F3C//ANP32B//AKAP11//DDX6//MDFIC//CMPK1//PIK3CB//SLC30A5//TBCA//ZNF655//DTD1//NRXN1//TMOD1//APPL1//CDK19//CNOT7//BHLHE40//TRIM8//DENND1B//MEOX2//SMU1//SLC2A4RG//SNURF//EI24//EPC2//TSHZ1//CALCOCO2//UBE4B//ZNF268//ADH4//KLF17//CPEB2//NCOA7//MTPN//UBXN2B//DYNLL2//ZNF782//SPRED1//CRISP1//ZNF800//ARX//DOK1//EMX2//EN1//CCNY//ZFP30//CPEB3//FOXJ3//FOXF2//PDZD2//MYT1L//DCUN1D4//CNOT6L//PTF1A//NUTM1//TES//PTPN22//GLRX//GSTM3//PURG//HOXB6//SPOPL//ZNF680//MACC1//IMPDH1//ZNF568//MAF//MGST1//MLLT6//MYBL1//MYD88//NAP1L3//NCK1//NEUROG1//NHLH2//OTX2//MEMO1//YTHDF2//VGLL1//ARMCX3//RASD1//POU3F2//EGLN1//XAF1//TET2//STRBP//BTBD7//SERTAD4//MAP3K7CL//DUSP22//ZNF630//CNOT6//RDH14//ZBTB26//MID1IP1//RFX4//DMRTA1//SAMSN1//FAM110C//SH3BGRL//GMNC//RFX7//BTF3//BTG1//TSC1//RHOXF2B//VBP1//ZNF3//ZNF134//ZNF208//ZNF227//PTP4A1//EVI5//DCTPP1//BHLHE41//NPEPL1//UBA5//FAM192A//CALM2//TSC22D4//RHOXF2//NFATC2IP//DGKE//ZIC5//BLZF1//KLF7//CBL//AKAP4//AIMP1//PLAA//QKI//PHF14//ST18// |
| GO:0005901 | caveola | Cellular component | 9 | 78 | 596 | 18698 | 3.61990191017037 | 0.000823046081817871 | 0.0785551760312835 | 3.0845758482398 | 0.0151006711409396 | CBL//SPRED1//EFNA5//LRP6//NOS1//BMPR2//TGFBR2//LRP8//AKAP6// |
| GO:0044853 | plasma membrane raft | Cellular component | 10 | 98 | 596 | 18698 | 3.20127379810985 | 0.00113446702710789 | 0.0923114283332708 | 2.94520812221465 | 0.0167785234899329 | SPRED1//EFNA5//LRP6//NOS1//BMPR2//TGFBR2//LRP8//AKAP6//CBL//CD8A// |
| GO:0005623 | cell | Cellular component | 560 | 16915 | 596 | 18698 | 1.03863970464244 | 0.00118210210904072 | 0.0923114283332708 | 2.92734500782226 | 0.939597315436242 | STX6//GPHN//RTN3//PROCR//TACC2//IQGAP2//SERINC3//TMED10//RAPGEF4//DNAJB4//AKAP11//SLC6A14//CHRM2//CLCN5//FRMD6//CCR1//RAET1E//DYNLL2//CSF2RA//SYT6//SH3D19//DGKH//SPRED1//ABHD3//DLG2//DPYSL2//DSC2//AGTR2//GPR183//EFNA5//HTR3D//EPS15//ESR1//ACSL1//CCNY//ZNRF2//ENPP4//SV2C//WDFY3//CLASP2//EFR3A//SULF1//SLC44A1//CD2AP//FLRT1//CADM2//SLC41A1//LRIG1//TES//STEAP2//SLC17A5//GJA1//GJB2//GLRB//DLL1//FAM126B//GRIK2//IGF1//IL1RAP//AR//KCNA4//KCNJ3//KCNJ10//KCNS2//TNFAIP8L3//LDLR//LRP6//MET//MGST1//MTM1//MYD88//NCK1//NEU1//NOS1//RAB9B//LGSN//CHMP3//RASD1//PIK3CB//CHIC1//PLP2//IL20RA//KCNK10//TREM1//PAQR5//PXK//RALGPS2//CPPED1//PPP3CB//TMEM63B//ENAH//PRKAR2B//CACNA2D3//PMEPA1//PTGFR//NLN//ARRDC3//STIM2//RAP2C//ENPP5//RP2//MSMO1//SAMSN1//ZMAT3//SLC1A1//SLC2A5//SLC6A6//SLC10A2//BMPR2//SNAP25//SOD1//BTC//TGFBR2//TNF//TRPC3//TSC1//C5AR1//FAM155A//WNT7A//YES1//LRP8//OR13A1//FZD3//ADAM12//CALM2//FZD6//CASP3//DGKE//OR6A2//LAMTOR3//CBL//SLC4A4//CD84//DIRAS3//DLG5//CD8A//MMGT1//SLIT2//RAB28//NRXN1//ITM2A//RAPGEF2//USP6NL//PHACTR2//TRERF1//ZNF217//TBL1XR1//FUT9//RAB12//HS3ST5//STX12//HEPACAM2//ARFIP1//RAB30//ST8SIA5//MAN1A1//ST8SIA3//RAB6B//RNF125//SYBU//CHST7//ACBD3//GNPTG//CHST1//BLZF1//TNKS//DSEL//NDST3//UBE4B//DCUN1D4//PPP1R15B//ZBTB18//SPO11//ADCYAP1//NCOA7//EREG//NYAP1//ARL5A//HSPB8//OSTF1//RNF180//IFNG//IL2//IL5//HPCAL4//ARL15//PRKAA1//CCL2//STK33//LONRF3//LONRF1//CDK6//PLEKHF2//ULK2//CALCOCO2//ATG16L1//PIGP//PIGA//FMR1//SYCP3//SYCP1//DYNC1LI2//FBXO28//MTBP//CLIP1//CFDP1//DCTN6//CENPO//CEBPB//PTGES3//ATR//WRNIP1//NABP1//ESCO2//BAHD1//MAF//H3F3C//IRF1//POU4F1//RFX3//SMARCD2//RUNX3//DDX6//TEX11//SYCE1//PCGF5//VPS37A//CETN1//FAM110C//CDC14A//MZT1//DCP2//PAN3//CNOT7//YTHDF2//CPEB1//RAI14//PAK6//KRT27//SNX5//ARHGAP12//SNRPN//DRAM1//RRAGD//SNX2//CMC4//MIEF2//CCDC58//PRSS35//IMMP1L//ELK3//AK4//MSRB3//GLRX//GLUD2//GPD1//COX8C//MACC1//JARID2//BOLA3//MDH1//DCAF8//MRPL35//XAF1//RDH14//METTL17//BNIP3//SOX4//UNG//DCTPP1//ABHD11//ZNF268//STRAP//EGLN3//KLF12//ARAP2//ADH4//MTPN//UBXN2B//CRKL//DENND1B//DCX//DOK1//PRSS55//RASGEF1A//RAB3GAP1//CDK19//OTUD3//DDHD2//DNAJC16//CNOT6L//LCLAT1//APPL1//PTPN22//KLHL3//KIF4B//NOB1//GSTA2//GSTM3//HPRT1//AGFG1//HSBP1//IMPDH1//IRF2//KLC1//SULT6B1//ARHGAP1//MBNL1//MAP3K1//NR3C2//MTRR//ARHGEF4//MEMO1//PEX5L//CMPK1//BRWD1//POLR2K//EGLN1//PPARG//MTMR10//SPHK2//KCMF1//MAP3K7CL//DUSP22//CNOT6//ZBTB4//MID1IP1//MPP4//RRM2//ATXN1//SRSF2//SH3BGR//SKP1//KRTAP4-11//MTMR9//SOS2//BTF3//THOP1//TMOD1//UBE2G1//VBP1//CA1//EVI5//SNIP1//UBA5//FOSL1//TRIM8//SBF2//KBTBD8//C1ORF198//EIF3B//LMLN//NAT1//CCT6A//NMI//AIMP1//DTD1//EI24//SOCS5//G3BP2//CDC27//ACBD5//APCDD1//LRP12//MEP1A//PCDHB10//PTPRG//SLC30A5//ACVR1//CD69//MED6//TSHZ1//ANP32B//ZMYND11//TCERG1//KLF17//CPEB2//RNF38//ZNF782//GADD45A//CRISP1//ZNF800//ARX//CC2D1B//EMX2//EN1//TFEC//ZFP30//CPEB3//FOXJ3//FOXF2//PDZD2//MYT1L//RCOR1//ZFPM2//PTF1A//NUTM1//INTS7//GATA2//ATAD2//PURG//MDFIC//HIVEP2//HOXB6//SPOPL//ZNF680//ZNF568//MDM4//MEOX2//MLLT6//MYB//MYBL1//MYOG//NAP1L3//NEUROD1//NEUROG1//NFIA//NFE2//NHLH2//NPAT//NR4A2//OTX2//VGLL1//ARMCX3//ARID4B//POU3F2//TET2//SMU1//UBE2W//STRBP//BTBD7//SERTAD4//SLC2A4RG//ZNF630//ZBTB26//MIER1//RBBP8//RFX4//DMRTA1//SH3BGRL//GMNC//RFX7//BTG1//RHOXF2B//ZNF3//ZNF131//ZNF134//ZNF208//ZNF227//PTP4A1//ZNF655//BHLHE41//NPEPL1//CARF//FAM192A//TSC22D4//ARID5B//RHOXF2//NFATC2IP//ZIC5//BHLHE40//KLF7//RTCA//AKAP4//SNURF//RPS6KA5//PLAA//QKI//PHF14//ST18//AKAP6//IFI44//SIVA1//STMN2//SLAIN1//REG3G//SLC30A7//EDN1//WDR47//NRBF2//RBM44//DESI2//COMMD2//ZFAND6//HECW2//RBM25//SPOCK1//TBCA//UBE3B//C5ORF30//RAD9B//PDIK1L//SCAF8//KDM2A//HBP1//HOXA3//HOXC8//HOXD1//KLF3//USP28//LDLRAD4//MED28//LCOR//CBFB//BTAF1//WDR20//RBM41//MTX3//BCL2A1//GPD2//MICU3//SLC25A53//COX7A2//FASTK//PABPC5//CLCN6//CNTNAP2//CDS1//VKORC1L1//SMIM14//FIBIN//SHISA2//FAM19A1//IER3IP1//RTN1//ELOVL7//PLA2G12A//CAMLG//ARL6IP1//ERP27//ARSK//SLC35D1//HSD11B1//PIGS//FUT2//HEATR5A//GLT6D1//CCDC126//OGN//C16ORF70//KIF13A//FAM92B//NTF3//NPTN//PSD//TMEM185A//NPNT//DBNDD2//MMRN1//NHLRC2//VEGFB//VWC2//EPC2//TDRD5//IDS//SESTD1//INHBB//CCL28//CCL8//CALCB// |
| GO:0030014 | CCR4-NOT complex | Cellular component | 4 | 16 | 596 | 18698 | 7.84312080536913 | 0.00136990961910114 | 0.0980626969006566 | 2.86330808482933 | 0.00671140939597315 | CNOT7//CPEB3//CNOT6L//CNOT6// |
| GO:0098588 | bounding membrane of organelle | Cellular component | 88 | 2040 | 596 | 18698 | 1.35332280563232 | 0.00193672657710278 | 0.127972933056253 | 2.7129316877152 | 0.147651006711409 | STX6//RTN3//FUT9//SERINC3//TMED10//CLCN5//RAB12//HS3ST5//STX12//HEPACAM2//GJA1//ARFIP1//RAB30//ST8SIA5//MAN1A1//ST8SIA3//RAB6B//RNF125//SYBU//CHST7//PMEPA1//ACBD3//GNPTG//CHST1//BLZF1//TNKS//DSEL//MMGT1//NDST3//PLEKHF2//SBF2//IQGAP2//RAB9B//SLC30A5//C5AR1//CLCN6//VPS37A//ZNRF2//APPL1//STEAP2//ARHGAP1//LDLR//MYD88//KIF13A//SNX2//LAMTOR3//WNT7A//EPS15//FUT2//USP6NL//CALCOCO2//ATG16L1//PHACTR2//MGST1//SLC2A5//SNAP25//RAP2C//ENPP4//SNX5//LRP6//LDLRAD4//CHMP3//CHRM2//ACSL1//PEX5L//ACBD5//MIEF2//SLC44A1//MTX3//BCL2A1//BNIP3//AKAP6//SLC17A5//NEU1//DRAM1//SPHK2//RDH14//SYT6//SV2C//DBNDD2//C16ORF70//WDFY3//RRAGD//NOS1//PSD//IER3IP1//ARMCX3//ARL6IP1// |
| GO:0005737 | cytoplasm | Cellular component | 401 | 11544 | 596 | 18698 | 1.0897752747585 | 0.00246834532958274 | 0.15145061700797 | 2.60759408117009 | 0.672818791946309 | SPOCK1//CLASP2//RAI14//FAM110C//TSC1//GRIK2//SOD1//SYBU//NOS1//RASD1//AKAP6//DDX6//ATG16L1//FMR1//STX6//RTN3//FUT9//SERINC3//TMED10//CLCN5//RAB12//HS3ST5//STX12//HEPACAM2//GJA1//ARFIP1//RAB30//ST8SIA5//MAN1A1//ST8SIA3//RAB6B//RNF125//CHST7//PMEPA1//ACBD3//GNPTG//CHST1//BLZF1//TNKS//DSEL//MMGT1//NDST3//NCK1//PPP1R15B//CCNY//PLEKHF2//ULK2//CALCOCO2//PIGP//PIGA//VPS37A//CHMP3//DCP2//PAN3//CNOT7//YTHDF2//CPEB1//DYNC1LI2//SLC17A5//LDLR//NEU1//DRAM1//ARRDC3//RRAGD//SNX2//CMC4//MIEF2//CCDC58//DYNLL2//PRSS35//IMMP1L//ELK3//AK4//ACSL1//SLC44A1//MSRB3//GLRX//GLUD2//GPD1//COX8C//MACC1//JARID2//BOLA3//MDH1//MGST1//DCAF8//MRPL35//XAF1//RDH14//METTL17//BNIP3//SOX4//UNG//ZNF217//DCTPP1//ABHD11//CDK6//TACC2//DCTN6//PTGES3//IQGAP2//ZNF268//RAPGEF4//DNAJB4//STRAP//AKAP11//EGLN3//KLF12//ARAP2//ADH4//MTPN//UBXN2B//CRKL//SYT6//SH3D19//SPRED1//DENND1B//DCX//DLG2//DOK1//DPYSL2//PRSS55//EPS15//ESR1//RASGEF1A//ZNRF2//RAB3GAP1//WDFY3//CDK19//EFR3A//OTUD3//DDHD2//DNAJC16//CNOT6L//LCLAT1//APPL1//TES//STEAP2//PTPN22//KLHL3//HSPB8//GJB2//SNX5//FAM126B//KIF4B//NOB1//GSTA2//GSTM3//HPRT1//AGFG1//HSBP1//KRT27//IMPDH1//IRF1//IRF2//AR//KLC1//TNFAIP8L3//SULT6B1//ARHGAP1//MBNL1//MAP3K1//NR3C2//MZT1//MTM1//MTRR//MYD88//ARHGEF4//MEMO1//RAB9B//PEX5L//CMPK1//PIK3CB//BRWD1//POLR2K//EGLN1//PPARG//MTMR10//PXK//CPPED1//PPP3CB//PRKAA1//ENAH//PRKAR2B//TRERF1//SPHK2//KCMF1//MAP3K7CL//DUSP22//CNOT6//ZBTB4//RAP2C//MID1IP1//MPP4//RRM2//CLIP1//ATXN1//SAMSN1//SRSF2//SH3BGR//NABP1//SKP1//KRTAP4-11//MTMR9//SNAP25//SOS2//BTF3//TGFBR2//THOP1//TMOD1//UBE2G1//VBP1//YES1//CA1//EVI5//CENPO//SNIP1//UBA5//FOSL1//TRIM8//SBF2//CASP3//KBTBD8//C1ORF198//DGKE//CDC14A//RUNX3//EIF3B//CBL//LMLN//NAT1//CCT6A//NMI//LONRF1//AIMP1//DTD1//ARHGAP12//EI24//SOCS5//RAPGEF2//USP6NL//G3BP2//CDC27//ACBD5//MTX3//BCL2A1//GPD2//MICU3//SLC25A53//COX7A2//NLN//FASTK//PABPC5//CLCN6//LAMTOR3//STMN2//DGKH//CNTNAP2//LRP6//PTP4A1//NRBF2//CDS1//VKORC1L1//SMIM14//PDZD2//SULF1//FIBIN//SHISA2//FAM19A1//IER3IP1//PLP2//STIM2//RTN1//MSMO1//ELOVL7//PLA2G12A//CAMLG//NRXN1//ARL6IP1//ERP27//ARSK//WNT7A//SLC35D1//FLRT1//RNF180//HSD11B1//PIGS//SLC30A7//ESCO2//FUT2//HEATR5A//GLT6D1//ATR//RP2//SLC30A5//CCDC126//ITM2A//OGN//C16ORF70//EDN1//C5AR1//CD2AP//CETN1//FAM92B//SV2C//NTF3//KIF13A//CALM2//AKAP4//NPNT//FZD6//CHRM2//DBNDD2//MED28//PHACTR2//MMRN1//IGF1//NHLRC2//VEGFB//ARMCX3//DSC2//DLL1//CHIC1//LDLRAD4//TDRD5//OSTF1//SLC2A5//IDS//AGTR2//INHBB//KCNS2//WRNIP1//STK33//TNF//PSD//ENPP4//GPHN//UBE4B//CEBPB//ANP32B//IFI44//SIVA1//SLAIN1//FRMD6//REG3G//CPEB2//GADD45A//TFEC//CPEB3//WDR47//ZFPM2//PTF1A//NUTM1//INTS7//GLRB//MDFIC//SPOPL//RBM44//MAF//MEOX2//MET//NEUROD1//NFE2//NPAT//NR4A2//DESI2//COMMD2//LGSN//ARID4B//ZFAND6//POU4F1//RALGPS2//SMU1//UBE2W//STRBP//TEX11//SLC2A4RG//PAK6//PTGFR//HECW2//MIER1//RBM25//SH3BGRL//BMPR2//SNRPN//TBCA//BTG1//ZNF655//NPEPL1//FZD3//TSC22D4//NFATC2IP//BHLHE40//UBE3B//C5ORF30//DLG5//RPS6KA5//SLIT2//RAB28//PLAA//QKI// |
| GO:0044464 | cell part | Cellular component | 557 | 16886 | 596 | 18698 | 1.03484976633675 | 0.00360978630369342 | 0.205110169883419 | 2.44251850720064 | 0.934563758389262 | STX6//GPHN//RTN3//PROCR//TACC2//IQGAP2//SERINC3//TMED10//RAPGEF4//DNAJB4//AKAP11//SLC6A14//CHRM2//CLCN5//FRMD6//CCR1//RAET1E//DYNLL2//CSF2RA//SYT6//SH3D19//DGKH//SPRED1//ABHD3//DLG2//DPYSL2//DSC2//AGTR2//GPR183//EFNA5//HTR3D//EPS15//ESR1//ACSL1//CCNY//ZNRF2//ENPP4//SV2C//WDFY3//CLASP2//EFR3A//SULF1//SLC44A1//CD2AP//FLRT1//CADM2//SLC41A1//LRIG1//TES//STEAP2//SLC17A5//GJA1//GJB2//GLRB//DLL1//FAM126B//GRIK2//IGF1//IL1RAP//AR//KCNA4//KCNJ3//KCNJ10//KCNS2//TNFAIP8L3//LDLR//LRP6//MET//MGST1//MTM1//MYD88//NCK1//NEU1//NOS1//RAB9B//LGSN//CHMP3//RASD1//PIK3CB//CHIC1//PLP2//IL20RA//KCNK10//TREM1//PAQR5//PXK//RALGPS2//CPPED1//PPP3CB//TMEM63B//ENAH//PRKAR2B//CACNA2D3//PMEPA1//PTGFR//NLN//ARRDC3//STIM2//RAP2C//ENPP5//RP2//MSMO1//SAMSN1//ZMAT3//SLC1A1//SLC2A5//SLC6A6//SLC10A2//BMPR2//SNAP25//SOD1//BTC//TGFBR2//TNF//TRPC3//TSC1//C5AR1//FAM155A//WNT7A//YES1//LRP8//OR13A1//FZD3//ADAM12//CALM2//FZD6//CASP3//DGKE//OR6A2//LAMTOR3//CBL//SLC4A4//CD84//DIRAS3//DLG5//CD8A//MMGT1//SLIT2//RAB28//NRXN1//ITM2A//RAPGEF2//USP6NL//PHACTR2//ADCYAP1//NCOA7//EREG//NYAP1//ARL5A//HSPB8//OSTF1//RNF180//IFNG//IL2//IL5//HPCAL4//ARL15//RNF125//PRKAA1//CCL2//STK33//LONRF3//LONRF1//LRP12//CNTNAP2//NPTN//AIMP1//RAB12//STX12//RAB30//RAB6B//RTN1//EVI5//HEPACAM2//KIF13A//SNX5//SNX2//FMR1//AGFG1//FAM92B//MDH1//ACVR1//EDN1//INHBB//RAB3GAP1//PPP1R15B//STMN2//KLC1//OTX2//MZT1//PSD//UBE4B//DCUN1D4//CDK6//CALCOCO2//CEBPB//ANP32B//IFI44//SIVA1//PTGES3//ZNF268//STRAP//EGLN3//ARAP2//SLAIN1//REG3G//CPEB2//SLC30A7//GADD45A//DDX6//DCP2//TFEC//CPEB3//WDR47//PDZD2//OTUD3//DDHD2//ZFPM2//CNOT6L//PTF1A//NUTM1//INTS7//APPL1//PTPN22//ARFIP1//GSTM3//CNOT7//MDFIC//NRBF2//HPRT1//HSBP1//SPOPL//MACC1//IMPDH1//IRF1//RBM44//ARHGAP1//MAF//MBNL1//MAP3K1//MEOX2//NEUROD1//NFE2//NPAT//NR4A2//DCAF8//DESI2//MEMO1//COMMD2//CMPK1//ARID4B//ZFAND6//POU4F1//EGLN1//MTMR10//SMU1//UBE2W//DRAM1//STRBP//TEX11//SLC2A4RG//PAK6//DUSP22//HECW2//MIER1//RBM25//RRAGD//ATXN1//SH3BGR//CPEB1//SH3BGRL//SKP1//MTMR9//SNRPN//BNIP3//SOX4//SPOCK1//TBCA//BTG1//UBE2G1//VBP1//PTP4A1//ZNF655//NPEPL1//UBA5//TSC22D4//NFATC2IP//BLZF1//BHLHE40//CDC14A//RUNX3//TNKS//AKAP4//UBE3B//C5ORF30//CCT6A//NMI//RPS6KA5//DTD1//PLAA//QKI//AKAP6//SOCS5//G3BP2//CDC27//FOXF2//RCOR1//GATA2//MYOG//NHLH2//POU3F2//TRERF1//RFX3//CNOT6//ARHGAP12//CD69//RNF38//NTF3//DCX//FOSL1//DBNDD2//C16ORF70//CETN1//NEUROG1//RRM2//GPD1//GPD2//KLHL3//KBTBD8//RAI14//FAM110C//PPARG//MTPN//TMEM185A//MICU3//SLC25A53//MRPL35//APCDD1//MEP1A//PCDHB10//PTPRG//SLC30A5//ARHGEF4//CLIP1//MIEF2//MTX3//BCL2A1//SYBU//JARID2//DYNC1LI2//FBXO28//MTBP//CMC4//CCDC58//PRSS35//IMMP1L//ELK3//AK4//MSRB3//GLRX//GLUD2//COX8C//BOLA3//XAF1//RDH14//METTL17//ACBD3//UNG//ZNF217//DCTPP1//ABHD11//MED6//TSHZ1//ZBTB18//ZMYND11//TCERG1//ADH4//KLF17//UBXN2B//ESCO2//ZNF782//CRISP1//ZNF800//ARX//DOK1//CC2D1B//EMX2//EN1//ZFP30//FOXJ3//MYT1L//CDK19//ATAD2//PURG//HIVEP2//HOXB6//ZNF680//ZNF568//MDM4//MLLT6//H3F3C//MYB//MYBL1//NAP1L3//NFIA//SYCP3//YTHDF2//VGLL1//ARMCX3//BRWD1//POLR2K//ATR//TET2//BTBD7//SERTAD4//WRNIP1//MAP3K7CL//ZNF630//ZBTB4//ZBTB26//MID1IP1//RBBP8//RFX4//DMRTA1//SRSF2//GMNC//NABP1//RFX7//BTF3//RHOXF2B//ZNF3//ZNF131//ZNF134//ZNF208//ZNF227//BHLHE41//TBL1XR1//SNIP1//CARF//FAM192A//ARID5B//PCGF5//RHOXF2//ZIC5//KLF7//RTCA//SNURF//ACBD5//PHF14//ST18//CDS1//VKORC1L1//SMIM14//LCLAT1//FIBIN//SHISA2//FAM19A1//MAN1A1//IER3IP1//PLEKHF2//ELOVL7//PLA2G12A//CAMLG//EI24//VPS37A//DENND1B//SPHK2//LDLRAD4//BTAF1//FUT9//FUT2//HEATR5A//GLT6D1//GNPTG//CCDC126//LMLN//TMOD1//ULK2//ATG16L1//DCTN6//KLF12//CRKL//PRSS55//RASGEF1A//DNAJC16//PAN3//KIF4B//NOB1//GSTA2//KRT27//IRF2//SULT6B1//NR3C2//MTRR//PEX5L//KCMF1//MPP4//KRTAP4-11//SOS2//THOP1//CA1//CENPO//TRIM8//SBF2//C1ORF198//EIF3B//NAT1//FASTK//PABPC5//ERP27//ARSK//OGN//HS3ST5//ST8SIA5//ST8SIA3//CHST7//CHST1//DSEL//NDST3//PIGP//PIGA//SLC35D1//ARL6IP1//HSD11B1//PIGS//NPNT//TDRD5//SPO11//BAHD1//RAD9B//PDIK1L//SCAF8//KDM2A//HBP1//HOXA3//HOXC8//HOXD1//KLF3//USP28//SMARCD2//MED28//LCOR//CBFB//WDR20//IDS//EPC2//CFDP1//SYCP1//RBM41//CLCN6//SESTD1//MMRN1//NHLRC2//VEGFB//SYCE1//COX7A2//VWC2// |
| GO:0000775 | chromosome, centromeric region | Cellular component | 14 | 194 | 596 | 18698 | 2.26399363453954 | 0.00382044553915565 | 0.205110169883419 | 2.41788598684911 | 0.023489932885906 | DYNC1LI2//CLASP2//FBXO28//MTBP//CLIP1//CEBPB//CFDP1//DCTN6//CENPO//ESCO2//FMR1//SYCP3//SYCP1//TNKS// |
| GO:0045202 | synapse | Cellular component | 42 | 880 | 596 | 18698 | 1.4973230628432 | 0.00608980408566861 | 0.307714218211137 | 2.21539667877626 | 0.0704697986577181 | DLG2//SPOCK1//NPTN//GLRB//CHRM2//CPEB3//FMR1//GRIK2//NOS1//PSD//CPEB1//BMPR2//BNIP3//LRP8//DLG5//SV2C//NTF3//SNAP25//STX6//SYT6//SLC17A5//DBNDD2//C16ORF70//ZNRF2//FOSL1//NRXN1//ADCYAP1//KCNA4//PRKAR2B//GPHN//FZD3//KCNJ10//WNT7A//RAB3GAP1//CPEB2//HTR3D//CADM2//VWC2//LRP6//ENAH//PLAA//RAPGEF2// |
| GO:0031981 | nuclear lumen | Cellular component | 155 | 4071 | 596 | 18698 | 1.1944816750992 | 0.00708186676945586 | 0.324127098657394 | 2.1498522478454 | 0.26006711409396 | ZBTB18//SPO11//MED6//CDK6//STX6//CEBPB//SIVA1//TACC2//PTGES3//ZMYND11//TCERG1//DNAJB4//STRAP//EGLN3//KLF12//VPS37A//CRKL//RAD9B//PDIK1L//RNF38//SH3D19//ESCO2//GADD45A//DCP2//CC2D1B//ELK3//ESR1//TFEC//SCAF8//BAHD1//KDM2A//RCOR1//FMR1//ZFPM2//SLC44A1//INTS7//GATA2//HSPB8//HBP1//GJA1//KIF4B//NOB1//ATAD2//NRBF2//HIVEP2//HOXA3//HOXC8//HOXD1//HSBP1//IRF1//IRF2//AR//JARID2//TNFAIP8L3//MBNL1//MDM4//NR3C2//MYB//MYOG//NEUROD1//NFIA//NFE2//NOS1//NPAT//NR4A2//DCAF8//KLF3//ARID4B//BRWD1//POLR2K//ATR//POU4F1//PPARG//UBE2W//PPP3CB//PRKAA1//TRERF1//USP28//ZBTB4//MIER1//RRAGD//RBBP8//RP2//ATXN1//SRSF2//ZMAT3//CPEB1//METTL17//NABP1//SKP1//BMPR2//SMARCD2//SNRPN//BNIP3//SOD1//SOX4//UNG//LDLRAD4//ZNF131//ZNF217//CENPO//TBL1XR1//SNIP1//CARF//MED28//ADAM12//FOSL1//CASP3//ARID5B//PCGF5//LCOR//CDC14A//RTCA//RUNX3//CBFB//TNKS//BTAF1//NMI//ACBD5//WDR20//RPS6KA5//CDC27//ANP32B//AKAP11//DDX6//WDFY3//MDFIC//CMPK1//PIK3CB//SLC30A5//TBCA//ZNF655//DTD1//NRXN1//WRNIP1//RFX3//SYCP3//TEX11//SYCP1//SYCE1//H3F3C//APPL1//CDK19//CNOT7//BHLHE40//TRIM8//DENND1B//MEOX2//SMU1//SLC2A4RG//RBM25//SNURF//EPC2//RAI14//PAK6// |
| GO:0044422 | organelle part | Cellular component | 327 | 9321 | 596 | 18698 | 1.10061173837816 | 0.00716928390511117 | 0.324127098657394 | 2.14452422112299 | 0.548657718120805 | CDC14A//MRPL35//APPL1//NCK1//NOS1//WDFY3//TNKS//NRXN1//AKAP6//EI24//BNIP3//PLEKHF2//DDX6//ATG16L1//RAB28//GSTM3//AKAP4//C5ORF30//RP2//DLG5//EPS15//DYNLL2//PRKAR2B//TMOD1//CALM2//PPP3CB//NPNT//MTM1//DLG2//GRIK2//IQGAP2//TMED10//RAB9B//SLC30A5//C5AR1//ZNRF2//FLRT1//SNX5//CLIP1//FZD6//ULK2//WNT7A//GPD2//MICU3//SLC25A53//STX6//RTN3//FUT9//SERINC3//CLCN5//RAB12//HS3ST5//STX12//HEPACAM2//GJA1//ARFIP1//RAB30//ST8SIA5//MAN1A1//ST8SIA3//RAB6B//RNF125//SYBU//CHST7//PMEPA1//ACBD3//GNPTG//CHST1//BLZF1//DSEL//MMGT1//NDST3//MIEF2//ACSL1//SLC44A1//MTX3//MGST1//BCL2A1//SBF2//CLCN6//VPS37A//STEAP2//ARHGAP1//LDLR//MYD88//KIF13A//SNX2//LAMTOR3//IER3IP1//ARMCX3//PSD//RNF180//CMC4//NLN//SOD1//THOP1//OSTF1//IMPDH1//SULF1//CLASP2//SNAP25//C16ORF70//RAB3GAP1//CETN1//FASTK//PABPC5//AK4//ERP27//ARSK//OGN//KCMF1//FMR1//SYCP3//SYCP1//DYNC1LI2//FBXO28//MTBP//CFDP1//DCTN6//CENPO//PTGES3//SPO11//ATR//WRNIP1//NABP1//ESCO2//BAHD1//MAF//H3F3C//CEBPB//ESR1//IRF1//AR//POU4F1//RFX3//SMARCD2//RUNX3//TEX11//SYCE1//RAD9B//TRERF1//ZNF217//TBL1XR1//ZBTB18//RAI14//PAK6//SNRPN//AGFG1//MED6//CDK6//SIVA1//TACC2//ZMYND11//TCERG1//DNAJB4//STRAP//EGLN3//KLF12//CRKL//PDIK1L//RNF38//SH3D19//GADD45A//DCP2//CC2D1B//ELK3//TFEC//SCAF8//KDM2A//RCOR1//ZFPM2//INTS7//GATA2//HSPB8//HBP1//KIF4B//NOB1//ATAD2//NRBF2//HIVEP2//HOXA3//HOXC8//HOXD1//HSBP1//IRF2//JARID2//TNFAIP8L3//MBNL1//MDM4//NR3C2//MYB//MYOG//NEUROD1//NFIA//NFE2//NPAT//NR4A2//DCAF8//KLF3//ARID4B//BRWD1//POLR2K//PPARG//UBE2W//PRKAA1//USP28//ZBTB4//MIER1//RRAGD//RBBP8//ATXN1//SRSF2//ZMAT3//CPEB1//METTL17//SKP1//BMPR2//SOX4//UNG//LDLRAD4//ZNF131//SNIP1//CARF//MED28//ADAM12//FOSL1//CASP3//ARID5B//PCGF5//LCOR//RTCA//CBFB//BTAF1//NMI//ACBD5//WDR20//RPS6KA5//CDC27//RBM25//RBM41//ANP32B//AKAP11//MDFIC//CMPK1//PIK3CB//TBCA//ZNF655//DTD1//CDK19//CNOT7//BHLHE40//SMU1//COX7A2//FAM110C//MZT1//KRT27//PROCR//FAM92B//DDHD2//YTHDF2//PXK//YES1//EVI5//PTP4A1//KBTBD8//KLC1//DCX//DPYSL2//WDR47//MID1IP1//CCT6A//LRP8//TSC1//MTPN//CD2AP//HECW2//FUT2//USP6NL//PIGP//PIGA//ARL6IP1//CDS1//VKORC1L1//SMIM14//SLC35D1//LCLAT1//HSD11B1//SHISA2//PLP2//STIM2//RDH14//MSMO1//ELOVL7//PIGS//RTN1//EDN1//SYT6//CALCOCO2//CPPED1//IDS//NEU1//PEX5L//CHRM2//PHACTR2//LRP6//CHMP3//SLC2A5//RAP2C//ENPP4//EPC2//KRTAP4-11//SLC17A5//DRAM1//SPHK2//SV2C//DBNDD2//TRIM8//DENND1B//MEOX2//SLC2A4RG//SNURF//MMRN1//IGF1//NHLRC2//VEGFB//COX8C//IMMP1L// |
| GO:0044446 | intracellular organelle part | Cellular component | 319 | 9080 | 596 | 18698 | 1.1021830559087 | 0.00772855798032056 | 0.331941565254768 | 2.1119015306109 | 0.535234899328859 | SNX5//PLEKHF2//WDFY3//RNF180//CLIP1//BNIP3//AKAP6//CMC4//NLN//SOD1//THOP1//DYNC1LI2//CLASP2//FBXO28//MTBP//ESCO2//BAHD1//MAF//H3F3C//ZBTB18//SPO11//SNRPN//AGFG1//TNKS//MED6//CDK6//STX6//CEBPB//SIVA1//TACC2//PTGES3//ZMYND11//TCERG1//DNAJB4//STRAP//EGLN3//KLF12//VPS37A//CRKL//RAD9B//PDIK1L//RNF38//SH3D19//GADD45A//DCP2//CC2D1B//ELK3//ESR1//TFEC//SCAF8//KDM2A//RCOR1//FMR1//ZFPM2//SLC44A1//INTS7//GATA2//HSPB8//HBP1//GJA1//KIF4B//NOB1//ATAD2//NRBF2//HIVEP2//HOXA3//HOXC8//HOXD1//HSBP1//IRF1//IRF2//AR//JARID2//TNFAIP8L3//MBNL1//MDM4//NR3C2//MYB//MYOG//NEUROD1//NFIA//NFE2//NOS1//NPAT//NR4A2//DCAF8//KLF3//ARID4B//BRWD1//POLR2K//ATR//POU4F1//PPARG//UBE2W//PPP3CB//PRKAA1//TRERF1//USP28//ZBTB4//MIER1//RRAGD//RBBP8//RP2//ATXN1//SRSF2//ZMAT3//CPEB1//METTL17//NABP1//SKP1//BMPR2//SMARCD2//SOX4//UNG//LDLRAD4//ZNF131//ZNF217//CENPO//TBL1XR1//SNIP1//CARF//MED28//ADAM12//FOSL1//CASP3//ARID5B//PCGF5//LCOR//CDC14A//RTCA//RUNX3//CBFB//BTAF1//NMI//ACBD5//WDR20//RPS6KA5//CDC27//RBM25//ANP32B//AKAP11//DDX6//MDFIC//CMPK1//PIK3CB//SLC30A5//TBCA//ZNF655//DTD1//NRXN1//TMOD1//EI24//FASTK//PABPC5//AK4//CETN1//FAM110C//CALM2//ATG16L1//DDHD2//YTHDF2//PXK//YES1//EVI5//HEPACAM2//MZT1//PTP4A1//KBTBD8//DCX//LRP8//MTPN//DYNLL2//IQGAP2//CD2AP//RAB28//RTN3//FUT9//SERINC3//TMED10//CLCN5//RAB12//HS3ST5//STX12//ARFIP1//RAB30//ST8SIA5//MAN1A1//ST8SIA3//RAB6B//RNF125//SYBU//CHST7//PMEPA1//ACBD3//GNPTG//CHST1//BLZF1//DSEL//MMGT1//NDST3//OGN//WNT7A//PIGP//PIGA//ERP27//ARSK//CDS1//VKORC1L1//SMIM14//ACSL1//RAB3GAP1//SLC35D1//ARL6IP1//FLRT1//LCLAT1//HSD11B1//SHISA2//MGST1//IER3IP1//PLP2//STIM2//RDH14//MSMO1//ELOVL7//PIGS//SYT6//EPS15//ZNRF2//FZD6//ULK2//OSTF1//IMPDH1//KCMF1//SBF2//FUT2//KIF13A//USP6NL//CFDP1//DCTN6//RFX3//RBM41//SMU1//CALCOCO2//CLCN6//SLC17A5//NEU1//DRAM1//SPHK2//LAMTOR3//CPPED1//IDS//PEX5L//EDN1//PROCR//PRKAR2B//DLG5//HECW2//KLC1//MRPL35//RAB9B//C5AR1//CHRM2//LDLR//PHACTR2//SLC2A5//SNAP25//RAP2C//ENPP4//RTN1//STEAP2//PSD//MIEF2//MTX3//BCL2A1//GPD2//MICU3//SLC25A53//SULF1//C16ORF70//SYCP3//SYCP1//WRNIP1//TEX11//SYCE1//RAI14//PAK6//CDK19//CNOT7//BHLHE40//COX7A2//KRT27//FAM92B//DPYSL2//WDR47//MID1IP1//CCT6A//TSC1//APPL1//ARHGAP1//MYD88//SNX2//CHMP3//LRP6//TRIM8//DENND1B//MEOX2//SLC2A4RG//SNURF//COX8C//IMMP1L//MMRN1//IGF1//NHLRC2//VEGFB//KRTAP4-11//EPC2//SV2C//DBNDD2//ARMCX3// |
| GO:0045121 | membrane raft | Cellular component | 18 | 302 | 596 | 18698 | 1.86988310591582 | 0.0084514390152799 | 0.340798767434893 | 2.07306933801041 | 0.0302013422818792 | CD8A//LAMTOR3//SPRED1//EFNA5//LRP6//NOS1//BMPR2//TGFBR2//LRP8//AKAP6//CBL//SULF1//STX12//GJA1//DLL1//PRKAR2B//TNF//CASP3// |
| GO:0098857 | membrane microdomain | Cellular component | 18 | 303 | 596 | 18698 | 1.86371187454316 | 0.00872825714035814 | 0.340798767434893 | 2.0590724676221 | 0.0302013422818792 | SULF1//STX12//GJA1//DLL1//NOS1//PRKAR2B//TGFBR2//TNF//CASP3//CBL//CD8A//SPRED1//EFNA5//LRP6//BMPR2//LRP8//AKAP6//LAMTOR3// |
| GO:0005829 | cytosol | Cellular component | 186 | 5022 | 596 | 18698 | 1.16194382301765 | 0.00916721231812873 | 0.342375451359677 | 2.03776271001308 | 0.312080536912752 | CCT6A//RRM2//STX12//SNAP25//TSC1//CDK6//STX6//CALCOCO2//TACC2//DCTN6//PTGES3//IQGAP2//ZNF268//RAPGEF4//DNAJB4//STRAP//AKAP11//EGLN3//KLF12//ARAP2//CLCN5//ADH4//MTPN//VPS37A//UBXN2B//CRKL//DYNLL2//SYT6//SH3D19//SPRED1//DENND1B//DCX//DDX6//DCP2//DLG2//DYNC1LI2//DOK1//DPYSL2//RAB12//PRSS55//EPS15//ESR1//RASGEF1A//ZNRF2//RAB3GAP1//WDFY3//CDK19//CLASP2//EFR3A//OTUD3//DDHD2//FMR1//DNAJC16//CNOT6L//LCLAT1//MSRB3//PAN3//APPL1//RAI14//TES//STEAP2//PTPN22//KLHL3//HSPB8//SLC17A5//GJA1//GJB2//SNX5//ARFIP1//GLRX//GPD1//FAM126B//KIF4B//NOB1//GSTA2//GSTM3//CNOT7//HPRT1//AGFG1//HSBP1//KRT27//IMPDH1//IRF1//IRF2//AR//KLC1//TNFAIP8L3//SULT6B1//ARHGAP1//MAN1A1//MBNL1//MDH1//MAP3K1//NR3C2//MZT1//MTM1//MTRR//MYD88//NCK1//NOS1//ARHGEF4//DCAF8//MEMO1//RAB9B//YTHDF2//PEX5L//RAB6B//CHMP3//CMPK1//PIK3CB//BRWD1//POLR2K//EGLN1//PPARG//XAF1//MTMR10//PXK//ATG16L1//CPPED1//PPP3CB//PRKAA1//ENAH//PRKAR2B//TRERF1//SPHK2//KCMF1//MAP3K7CL//DUSP22//CNOT6//ZBTB4//RAP2C//MID1IP1//RRAGD//MPP4//CLIP1//ATXN1//SAMSN1//SRSF2//SH3BGR//CPEB1//NABP1//SKP1//KRTAP4-11//MTMR9//SNX2//SOD1//SOS2//BTF3//TGFBR2//THOP1//TMOD1//UBE2G1//VBP1//YES1//CA1//EVI5//DCTPP1//CENPO//SNIP1//UBA5//FOSL1//TRIM8//SBF2//CASP3//KBTBD8//C1ORF198//DGKE//CDC14A//RUNX3//TNKS//EIF3B//CBL//LMLN//NAT1//NMI//LONRF1//AIMP1//DTD1//ARHGAP12//EI24//SOCS5//RAPGEF2//ULK2//USP6NL//G3BP2//CDC27// |
| GO:0005794 | Golgi apparatus | Cellular component | 64 | 1501 | 596 | 18698 | 1.33766750577914 | 0.0102830226377769 | 0.346166262143388 | 1.98787920810769 | 0.10738255033557 | STX6//RTN3//FUT9//SERINC3//TMED10//CLCN5//RAB12//HS3ST5//STX12//HEPACAM2//GJA1//ARFIP1//RAB30//ST8SIA5//MAN1A1//ST8SIA3//RAB6B//RNF125//SYBU//CHST7//PMEPA1//ACBD3//GNPTG//CHST1//BLZF1//TNKS//DSEL//MMGT1//NDST3//SULF1//OGN//WNT7A//CLASP2//SNAP25//C16ORF70//STEAP2//IER3IP1//FUT2//KIF13A//USP6NL//STMN2//UBXN2B//SLC30A7//ESCO2//ESR1//RAB3GAP1//DDHD2//HEATR5A//CNTNAP2//GLT6D1//FIBIN//LDLR//LRP6//ATR//RP2//SLC30A5//YES1//EVI5//PLA2G12A//KBTBD8//CCDC126//AIMP1//ITM2A//EI24// |
| GO:0010008 | endosome membrane | Cellular component | 24 | 451 | 596 | 18698 | 1.66948912930252 | 0.0103066005324459 | 0.346166262143388 | 1.98688455619582 | 0.0402684563758389 | VPS37A//CHMP3//SNX5//EPS15//STX12//APPL1//LRP6//PMEPA1//SNX2//LDLRAD4//PLEKHF2//MMGT1//LDLR//RAB12//RAP2C//LAMTOR3//PSD//CLCN5//CLCN6//ZNRF2//STEAP2//ARHGAP1//MYD88//KIF13A// |
| GO:0044444 | cytoplasmic part | Cellular component | 335 | 9626 | 596 | 18698 | 1.09181195503788 | 0.0105031466738979 | 0.346166262143388 | 1.97868056965577 | 0.562080536912752 | NCK1//PPP1R15B//CCNY//STX12//PLEKHF2//ULK2//ATG16L1//GJA1//CMC4//MIEF2//CCDC58//DYNLL2//DDX6//PRSS35//IMMP1L//ELK3//AK4//ACSL1//SLC44A1//MSRB3//RAI14//GLRX//GLUD2//GPD1//COX8C//MACC1//JARID2//BOLA3//MDH1//MGST1//NOS1//DCAF8//MRPL35//XAF1//RDH14//METTL17//ACBD3//BNIP3//SOD1//SOX4//UNG//ZNF217//DCTPP1//ABHD11//CDK6//STX6//CALCOCO2//TACC2//DCTN6//PTGES3//IQGAP2//ZNF268//RAPGEF4//DNAJB4//STRAP//AKAP11//EGLN3//KLF12//ARAP2//CLCN5//ADH4//MTPN//VPS37A//UBXN2B//CRKL//SYT6//SH3D19//SPRED1//DENND1B//DCX//DCP2//DLG2//DYNC1LI2//DOK1//DPYSL2//RAB12//PRSS55//EPS15//ESR1//RASGEF1A//ZNRF2//RAB3GAP1//WDFY3//CDK19//CLASP2//EFR3A//OTUD3//DDHD2//FMR1//DNAJC16//CNOT6L//LCLAT1//PAN3//APPL1//TES//STEAP2//PTPN22//KLHL3//HSPB8//SLC17A5//GJB2//SNX5//ARFIP1//FAM126B//KIF4B//NOB1//GSTA2//GSTM3//CNOT7//HPRT1//AGFG1//HSBP1//KRT27//IMPDH1//IRF1//IRF2//AR//KLC1//TNFAIP8L3//SULT6B1//ARHGAP1//MAN1A1//MBNL1//MAP3K1//NR3C2//MZT1//MTM1//MTRR//MYD88//ARHGEF4//MEMO1//RAB9B//YTHDF2//PEX5L//RAB6B//CHMP3//CMPK1//PIK3CB//BRWD1//POLR2K//EGLN1//PPARG//MTMR10//PXK//CPPED1//PPP3CB//PRKAA1//ENAH//PRKAR2B//TRERF1//SPHK2//KCMF1//MAP3K7CL//DUSP22//CNOT6//ZBTB4//RAP2C//MID1IP1//RRAGD//MPP4//RRM2//CLIP1//ATXN1//SAMSN1//SRSF2//SH3BGR//CPEB1//NABP1//SKP1//KRTAP4-11//MTMR9//SNAP25//SNX2//SOS2//BTF3//TGFBR2//THOP1//TMOD1//TSC1//UBE2G1//VBP1//YES1//CA1//EVI5//CENPO//SNIP1//UBA5//FOSL1//TRIM8//SBF2//CASP3//KBTBD8//C1ORF198//DGKE//CDC14A//RUNX3//TNKS//EIF3B//CBL//LMLN//NAT1//CCT6A//NMI//LONRF1//AIMP1//DTD1//ARHGAP12//EI24//SOCS5//RAPGEF2//USP6NL//G3BP2//CDC27//RTN3//CDS1//TMED10//VKORC1L1//SMIM14//PDZD2//SULF1//FIBIN//SHISA2//LRP6//FAM19A1//IER3IP1//PLP2//STIM2//RTN1//MSMO1//PTP4A1//ELOVL7//PLA2G12A//CAMLG//NRXN1//FUT9//STMN2//SLC30A7//ESCO2//FUT2//HEPACAM2//HEATR5A//CNTNAP2//GLT6D1//LDLR//ATR//RP2//SLC30A5//GNPTG//BLZF1//CCDC126//MMGT1//ITM2A//GPD2//DSC2//FLRT1//DLL1//NRBF2//NTF3//CHIC1//SYBU//SERINC3//AGTR2//CD2AP//INHBB//KCNS2//RASD1//WRNIP1//STK33//AKAP4//AKAP6//NPNT//DGKH//ARRDC3//EDN1//TDRD5//ACBD5//NLN//FASTK//PABPC5//HS3ST5//RAB30//ST8SIA5//ST8SIA3//RNF125//CHST7//PMEPA1//CHST1//DSEL//NDST3//OGN//WNT7A//PIGP//PIGA//ERP27//ARSK//SLC35D1//ARL6IP1//RNF180//HSD11B1//PIGS//FZD6//OSTF1//CALM2//CETN1//FAM92B//FAM110C//KIF13A//NEU1//DRAM1//TNF//CLCN6//LAMTOR3//IDS//SV2C//IGF1//C5AR1//CHRM2//PHACTR2//SLC2A5//ENPP4//MED28//MTX3//BCL2A1//MICU3//SLC25A53//GRIK2//COX7A2//C16ORF70//LDLRAD4//PSD//MMRN1//NHLRC2//VEGFB//DBNDD2//ARMCX3// |
| GO:0005654 | nucleoplasm | Cellular component | 133 | 3472 | 596 | 18698 | 1.20176851050011 | 0.010880662488791 | 0.346166262143388 | 1.96334466102874 | 0.223154362416107 | TRERF1//ZNF217//TBL1XR1//POLR2K//BAHD1//FMR1//NPAT//APPL1//MED6//CDK19//MED28//INTS7//CNOT7//USP28//ZBTB4//RP2//BHLHE40//CDC14A//TNKS//WDFY3//NFE2//ATR//TRIM8//DENND1B//GADD45A//HBP1//AR//MEOX2//NR4A2//SMU1//PRKAA1//SLC2A4RG//RBM25//SRSF2//SNURF//EPC2//JARID2//ATXN1//CDK6//STX6//CEBPB//SIVA1//TACC2//PTGES3//ZMYND11//TCERG1//DNAJB4//STRAP//EGLN3//KLF12//VPS37A//CRKL//RAD9B//PDIK1L//RNF38//SH3D19//ESCO2//DCP2//CC2D1B//ELK3//ESR1//TFEC//SCAF8//KDM2A//RCOR1//ZFPM2//SLC44A1//GATA2//HSPB8//GJA1//KIF4B//NOB1//ATAD2//NRBF2//HIVEP2//HOXA3//HOXC8//HOXD1//HSBP1//IRF1//IRF2//TNFAIP8L3//MBNL1//MDM4//NR3C2//MYB//MYOG//NEUROD1//NFIA//NOS1//DCAF8//KLF3//ARID4B//BRWD1//POU4F1//PPARG//UBE2W//PPP3CB//MIER1//RRAGD//RBBP8//ZMAT3//CPEB1//METTL17//NABP1//SKP1//BMPR2//SMARCD2//SNRPN//BNIP3//SOD1//SOX4//UNG//LDLRAD4//ZNF131//CENPO//SNIP1//CARF//ADAM12//FOSL1//CASP3//ARID5B//PCGF5//LCOR//RTCA//RUNX3//CBFB//BTAF1//NMI//ACBD5//WDR20//RPS6KA5//CDC27// |
| GO:0098589 | membrane region | Cellular component | 18 | 314 | 596 | 18698 | 1.79842260505279 | 0.0122804447845557 | 0.372791550570302 | 1.91078590322906 | 0.0302013422818792 | SULF1//STX12//GJA1//DLL1//NOS1//PRKAR2B//TGFBR2//TNF//CASP3//CBL//SPRED1//EFNA5//LRP6//BMPR2//LRP8//AKAP6//CD8A//LAMTOR3// |
| GO:0042734 | presynaptic membrane | Cellular component | 7 | 79 | 596 | 18698 | 2.77984028544729 | 0.0129330730869856 | 0.372791550570302 | 1.88829826814787 | 0.011744966442953 | ZNRF2//FMR1//NPTN//GRIK2//SNAP25//FOSL1//NRXN1// |
| GO:0031901 | early endosome membrane | Cellular component | 10 | 138 | 596 | 18698 | 2.27336834938236 | 0.01301949536334 | 0.372791550570302 | 1.88540584872974 | 0.0167785234899329 | EPS15//STX12//APPL1//SNX5//LRP6//PMEPA1//SNX2//LDLRAD4//PLEKHF2//MMGT1// |
| GO:0098791 | Golgi subcompartment | Cellular component | 38 | 819 | 596 | 18698 | 1.455621932132 | 0.0134639748605567 | 0.373082400168329 | 1.87082670793031 | 0.063758389261745 | STX6//RTN3//FUT9//SERINC3//TMED10//CLCN5//RAB12//HS3ST5//STX12//HEPACAM2//GJA1//ARFIP1//RAB30//ST8SIA5//MAN1A1//ST8SIA3//RAB6B//RNF125//SYBU//CHST7//PMEPA1//ACBD3//GNPTG//CHST1//BLZF1//TNKS//DSEL//MMGT1//NDST3//SULF1//CLASP2//SNAP25//C16ORF70//KIF13A//USP6NL//FUT2//STEAP2//IER3IP1// |
| GO:0099572 | postsynaptic specialization | Cellular component | 14 | 229 | 596 | 18698 | 1.91796840655315 | 0.0154103735592349 | 0.404831435212535 | 1.81218683352376 | 0.023489932885906 | DLG2//CPEB3//FMR1//NPTN//GRIK2//NOS1//PSD//CPEB1//BMPR2//BNIP3//SPOCK1//LRP8//DLG5//GPHN// |
| GO:0098590 | plasma membrane region | Cellular component | 47 | 1069 | 596 | 18698 | 1.37933275155229 | 0.0160055375133159 | 0.404831435212535 | 1.795729736381 | 0.0788590604026846 | EPS15//MET//BMPR2//DLG2//SLC41A1//KCNJ10//LDLR//C5AR1//SLC4A4//GJA1//DLL1//PRKAA1//SLC30A5//SLC1A1//SLC2A5//SLC10A2//FZD3//FZD6//RAPGEF2//PSD//CD8A//RP2//SLC6A14//CLASP2//ARHGEF4//SPRED1//EFNA5//LRP6//NOS1//TGFBR2//LRP8//AKAP6//ZNRF2//FMR1//NPTN//GRIK2//SNAP25//FOSL1//NRXN1//GPHN//CHRM2//CPEB3//GLRB//CPEB1//DLG5//CNTNAP2//CBL// |
| GO:0098687 | chromosomal region | Cellular component | 19 | 347 | 596 | 18698 | 1.71780167495116 | 0.0160235958058512 | 0.404831435212535 | 1.79524001862601 | 0.0318791946308725 | FMR1//SYCP3//SYCP1//TNKS//PTGES3//SPO11//ESCO2//CEBPB//ATR//WRNIP1//NABP1//DYNC1LI2//CLASP2//FBXO28//MTBP//CLIP1//CFDP1//DCTN6//CENPO// |
| GO:0044440 | endosomal part | Cellular component | 25 | 496 | 596 | 18698 | 1.5812743559212 | 0.0166979232828547 | 0.40648042067318 | 1.77733753860567 | 0.0419463087248322 | CLCN5//CLCN6//VPS37A//ZNRF2//APPL1//STEAP2//ARHGAP1//LDLR//MYD88//PMEPA1//KIF13A//SNX2//LAMTOR3//SNX5//RRAGD//EPS15//STX12//LRP6//LDLRAD4//PLEKHF2//MMGT1//CHMP3//RAB12//RAP2C//PSD// |
| GO:0032279 | asymmetric synapse | Cellular component | 14 | 232 | 596 | 18698 | 1.89316709095117 | 0.0170661512299671 | 0.40648042067318 | 1.76786441022985 | 0.023489932885906 | DLG2//CPEB3//FMR1//NPTN//GRIK2//NOS1//PSD//CPEB1//BMPR2//BNIP3//SPOCK1//LRP8//DLG5//CHRM2// |
| GO:0000139 | Golgi membrane | Cellular component | 34 | 729 | 596 | 18698 | 1.46318851787408 | 0.0175084698078087 | 0.40648042067318 | 1.75675180840337 | 0.0570469798657718 | FUT9//FUT2//STX6//ARFIP1//KIF13A//USP6NL//STEAP2//IER3IP1//RTN3//SERINC3//TMED10//CLCN5//RAB12//HS3ST5//STX12//HEPACAM2//GJA1//RAB30//ST8SIA5//MAN1A1//ST8SIA3//RAB6B//RNF125//SYBU//CHST7//PMEPA1//ACBD3//GNPTG//CHST1//BLZF1//TNKS//DSEL//MMGT1//NDST3// |
| GO:0098984 | neuron to neuron synapse | Cellular component | 14 | 235 | 596 | 18698 | 1.86899900042839 | 0.0188543070334513 | 0.426206572150912 | 1.72458942491727 | 0.023489932885906 | CHRM2//DLG2//CPEB3//FMR1//NPTN//GRIK2//NOS1//PSD//CPEB1//BMPR2//BNIP3//SPOCK1//LRP8//DLG5// |
| GO:0044456 | synapse part | Cellular component | 34 | 740 | 596 | 18698 | 1.44143841828406 | 0.0212900559570929 | 0.468927129926739 | 1.67182319709504 | 0.0570469798657718 | SV2C//NTF3//SNAP25//SYT6//SLC17A5//DBNDD2//C16ORF70//FMR1//FZD3//KCNJ10//WNT7A//RAB3GAP1//PSD//GPHN//ZNRF2//NPTN//GRIK2//FOSL1//NRXN1//CHRM2//DLG2//CPEB3//GLRB//CPEB1//DLG5//STX6//ADCYAP1//KCNA4//NOS1//PRKAR2B//BMPR2//BNIP3//SPOCK1//LRP8// |
| GO:0000407 | phagophore assembly site | Cellular component | 4 | 34 | 596 | 18698 | 3.69088037899724 | 0.0222288515431934 | 0.477364586890078 | 1.65308297460816 | 0.00671140939597315 | ATG16L1//ULK2//STX12//PLEKHF2// |
| GO:0031410 | cytoplasmic vesicle | Cellular component | 89 | 2279 | 596 | 18698 | 1.22516498758728 | 0.024063084496524 | 0.504150965427174 | 1.61864870393051 | 0.149328859060403 | STMN2//CLCN5//CRKL//DGKH//RAB12//STEAP2//SNX5//ARRDC3//CLIP1//SNX2//TMED10//EDN1//PLEKHF2//AIMP1//CD2AP//HEATR5A//RAB9B//RAPGEF2//STX6//CNTNAP2//APPL1//GJA1//LDLR//LRP6//PTP4A1//MMGT1//DYNC1LI2//MTM1//CHMP3//TNF//ARHGAP1//DDHD2//IER3IP1//IGF1//STX12//DENND1B//SLC30A5//SV2C//NTF3//SNAP25//SOD1//EPS15//PMEPA1//LDLRAD4//VPS37A//IQGAP2//C5AR1//OSTF1//IMPDH1//WNT7A//SYT6//SLC17A5//DBNDD2//C16ORF70//CHRM2//PHACTR2//MMRN1//NHLRC2//VEGFB//SLC2A5//LAMTOR3//NEU1//MGST1//CPPED1//RAP2C//ENPP4//KCMF1//CLCN6//ZNRF2//MYD88//KIF13A//FLRT1//FZD6//ULK2//RRAGD//PSD//CALCOCO2//SLC30A7//SPRED1//DSC2//DLL1//NRBF2//AGFG1//KLC1//RAB6B//CHIC1//SYBU//RP2//USP6NL// |
| GO:0097708 | intracellular vesicle | Cellular component | 89 | 2282 | 596 | 18698 | 1.22355434124076 | 0.0247827873951829 | 0.506867008868146 | 1.60584984879663 | 0.149328859060403 | CALCOCO2//SLC30A7//SPRED1//DSC2//FLRT1//DLL1//NRBF2//AGFG1//KLC1//LRP6//NTF3//RAB6B//CHMP3//CHIC1//SYBU//RP2//SOD1//USP6NL//STMN2//CLCN5//CRKL//DGKH//RAB12//STEAP2//SNX5//ARRDC3//CLIP1//SNX2//TMED10//EDN1//PLEKHF2//AIMP1//CD2AP//HEATR5A//RAB9B//RAPGEF2//STX6//CNTNAP2//APPL1//GJA1//LDLR//PTP4A1//MMGT1//DYNC1LI2//MTM1//TNF//ARHGAP1//DDHD2//IER3IP1//SV2C//SNAP25//IGF1//STX12//DENND1B//SLC30A5//EPS15//PMEPA1//LDLRAD4//VPS37A//IQGAP2//C5AR1//OSTF1//IMPDH1//WNT7A//SYT6//SLC17A5//DBNDD2//C16ORF70//CHRM2//PHACTR2//MMRN1//NHLRC2//VEGFB//SLC2A5//LAMTOR3//NEU1//MGST1//CPPED1//RAP2C//ENPP4//KCMF1//CLCN6//ZNRF2//MYD88//KIF13A//RRAGD//PSD//FZD6//ULK2// |
| GO:0044428 | nuclear part | Cellular component | 162 | 4435 | 596 | 18698 | 1.14596218306183 | 0.0256383670882072 | 0.512171100669069 | 1.59110963855899 | 0.271812080536913 | ZBTB18//SPO11//SNRPN//WDFY3//RNF180//CLIP1//BNIP3//AKAP6//AGFG1//TNKS//MED6//CDK6//STX6//CEBPB//SIVA1//TACC2//PTGES3//ZMYND11//TCERG1//DNAJB4//STRAP//EGLN3//KLF12//VPS37A//CRKL//RAD9B//PDIK1L//RNF38//SH3D19//ESCO2//GADD45A//DCP2//CC2D1B//ELK3//ESR1//TFEC//SCAF8//BAHD1//KDM2A//RCOR1//FMR1//ZFPM2//SLC44A1//INTS7//GATA2//HSPB8//HBP1//GJA1//KIF4B//NOB1//ATAD2//NRBF2//HIVEP2//HOXA3//HOXC8//HOXD1//HSBP1//IRF1//IRF2//AR//JARID2//TNFAIP8L3//MBNL1//MDM4//NR3C2//MYB//MYOG//NEUROD1//NFIA//NFE2//NOS1//NPAT//NR4A2//DCAF8//KLF3//ARID4B//BRWD1//POLR2K//ATR//POU4F1//PPARG//UBE2W//PPP3CB//PRKAA1//TRERF1//USP28//ZBTB4//MIER1//RRAGD//RBBP8//RP2//ATXN1//SRSF2//ZMAT3//CPEB1//METTL17//NABP1//SKP1//BMPR2//SMARCD2//SOD1//SOX4//UNG//LDLRAD4//ZNF131//ZNF217//CENPO//TBL1XR1//SNIP1//CARF//MED28//ADAM12//FOSL1//CASP3//ARID5B//PCGF5//LCOR//CDC14A//RTCA//RUNX3//CBFB//BTAF1//NMI//ACBD5//WDR20//RPS6KA5//CDC27//RBM25//ANP32B//AKAP11//DDX6//MDFIC//CMPK1//PIK3CB//SLC30A5//TBCA//ZNF655//DTD1//NRXN1//TMOD1//EI24//RBM41//SMU1//CDK19//CNOT7//BHLHE40//RAI14//PAK6//WRNIP1//RFX3//SYCP3//TEX11//SYCP1//SYCE1//APPL1//H3F3C//TRIM8//DENND1B//MEOX2//SLC2A4RG//SNURF//EPC2// |
| GO:0045178 | basal part of cell | Cellular component | 5 | 53 | 596 | 18698 | 2.95966822844118 | 0.0264235361535141 | 0.515859489906105 | 1.57800906298215 | 0.00838926174496644 | EPS15//MET//BMPR2//CLASP2//EDN1// |
| GO:0031312 | extrinsic component of organelle membrane | Cellular component | 4 | 37 | 596 | 18698 | 3.39161980772719 | 0.0293914942230826 | 0.561050967502843 | 1.53177833443287 | 0.00671140939597315 | SNX5//PLEKHF2//WDFY3//PSD// |
| GO:0014069 | postsynaptic density | Cellular component | 13 | 227 | 596 | 18698 | 1.79666203471011 | 0.0305285353086934 | 0.570087213699296 | 1.51529403168599 | 0.0218120805369128 | DLG2//GRIK2//CPEB3//FMR1//NPTN//NOS1//PSD//CPEB1//BMPR2//BNIP3//SPOCK1//LRP8//DLG5// |
| GO:0005768 | endosome | Cellular component | 38 | 878 | 596 | 18698 | 1.35780679090673 | 0.0349255809086989 | 0.622270350716047 | 1.45685636163914 | 0.063758389261745 | STX6//CNTNAP2//APPL1//STEAP2//GJA1//LDLR//LRP6//ARRDC3//PTP4A1//MMGT1//DYNC1LI2//MTM1//CHMP3//RAPGEF2//TNF//ARHGAP1//CLIP1//EPS15//STX12//SNX5//PMEPA1//SNX2//LDLRAD4//PLEKHF2//VPS37A//RAB12//RAP2C//CLCN5//CLCN6//ZNRF2//MYD88//KIF13A//LAMTOR3//RRAGD//PSD//STMN2//CRKL//DGKH// |
| GO:0098793 | presynapse | Cellular component | 19 | 379 | 596 | 18698 | 1.57276301110304 | 0.0354595019731977 | 0.622270350716047 | 1.45026736833496 | 0.0318791946308725 | SV2C//NTF3//SNAP25//ZNRF2//FMR1//NPTN//GRIK2//FOSL1//NRXN1//STX6//ADCYAP1//CHRM2//FZD3//SYT6//SLC17A5//DBNDD2//C16ORF70//KCNJ10//WNT7A// |
| GO:0001891 | phagocytic cup | Cellular component | 3 | 23 | 596 | 18698 | 4.09206302888824 | 0.0355311333668426 | 0.622270350716047 | 1.44939093922851 | 0.00503355704697987 | SNX5//TNF//ARHGAP12// |
| GO:0044431 | Golgi apparatus part | Cellular component | 40 | 935 | 596 | 18698 | 1.34213831963536 | 0.0362206257692693 | 0.622270350716047 | 1.44104405082503 | 0.0671140939597315 | STX6//RTN3//FUT9//SERINC3//TMED10//CLCN5//RAB12//HS3ST5//STX12//HEPACAM2//GJA1//ARFIP1//RAB30//ST8SIA5//MAN1A1//ST8SIA3//RAB6B//RNF125//SYBU//CHST7//PMEPA1//ACBD3//GNPTG//CHST1//BLZF1//TNKS//DSEL//MMGT1//NDST3//OGN//WNT7A//FUT2//KIF13A//USP6NL//STEAP2//IER3IP1//SULF1//CLASP2//SNAP25//C16ORF70// |
| GO:0044224 | juxtaparanode region of axon | Cellular component | 2 | 10 | 596 | 18698 | 6.2744966442953 | 0.0385243399374929 | 0.637824038218132 | 1.41426479359489 | 0.00335570469798658 | DLG2//CNTNAP2// |
| GO:0034705 | potassium channel complex | Cellular component | 7 | 99 | 596 | 18698 | 2.21825638939733 | 0.0388730338599676 | 0.637824038218132 | 1.41035156337631 | 0.011744966442953 | DLG2//CNTNAP2//KCNA4//KCNJ3//KCNS2//SNAP25//GRIK2// |
| GO:0005769 | early endosome | Cellular component | 17 | 334 | 596 | 18698 | 1.59680303821886 | 0.0398764048928898 | 0.637824038218132 | 1.39928400295717 | 0.0285234899328859 | EPS15//STX12//APPL1//SNX5//LRP6//PMEPA1//SNX2//LDLRAD4//PLEKHF2//MMGT1//STX6//CNTNAP2//STEAP2//GJA1//LDLR//ARRDC3//PTP4A1// |
| GO:0000795 | synaptonemal complex | Cellular component | 4 | 41 | 596 | 18698 | 3.06073007038795 | 0.0408385589080294 | 0.637824038218132 | 1.38892959149534 | 0.00671140939597315 | SYCP3//TEX11//SYCP1//SYCE1// |
| GO:0099086 | synaptonemal structure | Cellular component | 4 | 41 | 596 | 18698 | 3.06073007038795 | 0.0408385589080294 | 0.637824038218132 | 1.38892959149534 | 0.00671140939597315 | SYCP3//TEX11//SYCP1//SYCE1// |
| GO:0005815 | microtubule organizing center | Cellular component | 31 | 704 | 596 | 18698 | 1.38145877821843 | 0.0439139191334399 | 0.671324252816557 | 1.35739780220161 | 0.0520134228187919 | MZT1//CDK6//PROCR//DCTN6//CETN1//VPS37A//DYNLL2//DYNC1LI2//HEPACAM2//PRKAR2B//RRAGD//CLIP1//KIF13A//CALM2//PCGF5//CDC14A//CDC27//RP2//DLG5//RAB28//TNKS//FAM92B//TACC2//AKAP11//CLASP2//DDHD2//YTHDF2//PXK//FAM110C//YES1//EVI5// |
| GO:0034703 | cation channel complex | Cellular component | 12 | 217 | 596 | 18698 | 1.73488386478211 | 0.0458638126847885 | 0.671324252816557 | 1.3385298449585 | 0.0201342281879195 | VWC2//CALM2//SESTD1//AKAP6//DLG2//CNTNAP2//KCNA4//KCNJ3//KCNS2//SNAP25//GRIK2//NOS1// |
| GO:0016442 | RISC complex | Cellular component | 2 | 11 | 596 | 18698 | 5.70408785845027 | 0.0461095819745947 | 0.671324252816557 | 1.33620881503823 | 0.00335570469798658 | DDX6//DCP2// |
| GO:0031332 | RNAi effector complex | Cellular component | 2 | 11 | 596 | 18698 | 5.70408785845027 | 0.0461095819745947 | 0.671324252816557 | 1.33620881503823 | 0.00335570469798658 | DDX6//DCP2// |
| GO:0015630 | microtubule cytoskeleton | Cellular component | 47 | 1152 | 596 | 18698 | 1.27995374254288 | 0.0489916207877429 | 0.701396704277853 | 1.30987819255981 | 0.0788590604026846 | TACC2//AKAP11//CLASP2//DDHD2//YTHDF2//PXK//FAM110C//YES1//EVI5//HEPACAM2//MZT1//PTP4A1//KBTBD8//CDC27//DYNLL2//DCX//DYNC1LI2//DPYSL2//WDR47//KIF4B//KLC1//SYBU//MID1IP1//CLIP1//KIF13A//TBCA//CCT6A//LRP8//CDK6//PROCR//DCTN6//CETN1//VPS37A//PRKAR2B//RRAGD//CALM2//PCGF5//CDC14A//RP2//DLG5//RAB28//HECW2//TBL1XR1//TNKS//FAM92B//HOXC8//STRBP// |
